# Unexpected sounds induce a rapid inhibition of eye‐movement responses

**DOI:** 10.1111/psyp.14728

**Published:** 2024-12-17

**Authors:** Martin R. Vasilev, Zeynep G. Ozkan, Julie A. Kirkby, Antje Nuthmann, Fabrice B. R. Parmentier

**Affiliations:** ^1^ Department of Experimental Psychology University College London London UK; ^2^ Department of Methodology and ERI‐Lectura Universitat de València València Spain; ^3^ Department of Psychology Bournemouth University Bournemouth UK; ^4^ Department of Psychology Kiel University Kiel Germany; ^5^ Department of Psychology and Research Institute for Health Sciences (iUNICS) University of the Balearic Islands Palma Spain; ^6^ Balearic Islands Health Research Institute (IdISBa) Palma Spain; ^7^ School of Psychology University of Western Australia Perth Western Australia Australia

**Keywords:** anti‐saccade, inhibition, novelty distraction, pro‐saccade, saccades

## Abstract

Unexpected sounds have been shown to trigger a global and transient inhibition of motor responses. Recent evidence suggests that eye movements may also be inhibited in a similar way, but it is not clear how quickly unexpected sounds can affect eye‐movement responses. Additionally, little is known about whether they affect only voluntary saccades or also reflexive saccades. In this study, participants performed a pro‐saccade and an anti‐saccade task while the timing of sounds relative to stimulus onset was manipulated. Pro‐saccades are generally reflexive and stimulus‐driven, whereas anti‐saccades require the generation of a voluntary saccade in the opposite direction of a peripheral stimulus. Unexpected novel sounds inhibited the execution of both pro‐ and anti‐saccades compared to standard sounds, but the inhibition was stronger for anti‐saccades. Novel sounds affected response latencies as early as 150 ms before the peripheral cue to make a saccade, all the way to 25 ms after the cue to make a saccade. Interestingly, unexpected sounds also reduced anti‐saccade task errors, indicating that they aided inhibitory control. Overall, these results suggest that unexpected sounds yield a global and rapid inhibition of eye‐movement responses. This inhibition also helps suppress reflexive eye‐movement responses in favor of more voluntarily generated ones.

## Abstract 2

There is a growing understanding that unexpected sounds trigger global inhibition of motor actions. Previous work has suggested that unexpected sounds can also inhibit microsaccades and eye movements during active vision tasks. Here we show that unexpected sounds cause rapid inhibition of the oculomotor system within a window of at least 175 ms when programming both voluntary and reflexive simple saccades. Unexpected sounds also aid response inhibition, thus showing that their effect is inhibitory in nature.

## 1 INTRODUCTION

Novel or unexpected sounds that deviate from a repetitive sound sequence cause distraction in unrelated tasks (Berti, 2012; Dalton & Hughes, 2014; Escera et al., 1998; Horváth et al., 2008; Parmentier, 2014; Schröger, 1996; Schröger & Wolff, 1998). Novel sounds are distracting not due to their novelty per se, but because they violate predictions that another repeated sound will be presented (Parmentier et al., 2011). Specifically, the violation of expectations is thought to elicit an obligatory orientating response (Sokolov, 1963, 2001) toward the novel sound, which temporarily disengages attention from the task at hand and leads to distraction (Escera et al., 1998; Parmentier, 2014; Schröger, 1996; Schröger & Wolff, 1998).

Recent evidence has suggested that novel sounds also induce global inhibition of motor responses in addition to the attention‐orienting response (Wessel, 2017, 2018a, 2018b). For example, TMS stimulation of the cortical representation of task‐irrelevant muscles has shown that novel sounds lead to a reduction in motor‐evoked potentials (MEPs) some 150 ms after their presentation (Dutra et al., 2018; Iacullo et al., 2020; Wessel & Aron, 2013). Similar non‐selective inhibition of MEPs has also been observed following the successful stopping of actions (Badry et al., 2009; Cai et al., 2012; Majid et al., 2012), indicating that novel sounds may activate the same neural networks involved in action‐stopping. This is thought to occur via a fronto‐basal network that includes the right inferior frontal cortex (rIFC), the pre‐supplementary motor area (pre‐SMA), and the subthalamic nucleus (STN) (Wessel & Aron, 2013, 2017). This global inhibitory response may serve to stop ongoing actions in order to facilitate the attention‐orienting response and process the unexpected stimulus (Wessel, 2017, 2018a).

Interestingly, there is evidence that unexpected sounds may induce similar motor inhibition in eye‐movement planning. It is well‐established that remote visual distractors (Bompas & Sumner, 2009; Buonocore & McIntosh, 2012; Walker et al., 1997), as well as large transient displacements of visual information (Reingold & Stampe, 2000, 2004), lead to saccadic inhibition,^1^ that is, a decrease in the proportion of terminated fixations and, therefore, greater latency in making the next eye movement (see Buonocore & Hafed, 2023 for an overview). However, sounds can also cause such inhibition. For example, microsaccades (miniature eye movements occurring within a fixation) are inhibited by both a repeated standard sound (Rolfs et al., 2008) and pitch deviants (Kadosh & Bonneh, 2022b; Valsecchi & Turatto, 2009; Widmann et al., 2014). Additionally, the amount of inhibition is related to the perceived saliency of the unexpected sound. Environmental sounds with greater acoustical salience are associated with greater microsaccade inhibition (Zhao et al., 2019) and pitch deviants with larger acoustical deviation also show inhibition that is both larger in magnitude and earlier in its onset (Kadosh & Bonneh, 2022b).

Evidence from active vision tasks also shows similar results. For example, the presentation of a single‐pitched deviant sound during a scene‐viewing task leads to saccadic inhibition some from 90 to 150 ms after the sound onset (Graupner et al., 2007). Additionally, the presentation of a deviant or novel sound during sentence reading leads to an immediate increase in fixation durations (Rettie et al., 2024; Vasilev et al., 2019, 2021). This effect is largely constrained only to the fixation during which the sound is played and already disappears by the next fixation (though see Rettie et al., 2024). Furthermore, distraction does not appear to be related to the processing of text meaning but occurs even when scanning meaningless letter strings (Vasilev et al., 2023).

Overall, these findings suggest that unexpected sounds inhibit the planning stages of the next eye movement. This may occur either due to a brief pause in saccade programming or due to a slower accumulation of the neural signals leading up to a saccade. Despite this, once the saccade is executed, there is no evidence of inhibition as key saccadic metrics such as amplitude, duration, and velocity are unaffected (Vasilev et al., 2021). Therefore, the available evidence suggests that only the planning, but not the execution, of saccades is inhibited. This may be because saccades are ballistic and their velocity and duration are not under conscious control (Leigh & Zee, 1999).

The inhibition of saccade planning by unexpected sounds could be explained by Wessel and Aron's (2013, 2017) global motor inhibition account as the planning of motor actions may be briefly put on hold to allow for processing of the unexpected sound stimulus. Interestingly, data from MEPs indicate that inhibition is observed at 150 ms after the sound's onset, but no inhibition occurs in the 25 ms window before or after that (Dutra et al., 2018; Iacullo et al., 2020; Wessel & Aron, 2013). This suggests that the inhibition is relatively transient, even if most studies have used a limited number of sound onsets. However, evidence from frontal beta‐bursts (Tatz et al., 2023) and isometric force exertion (Novembre et al., 2018) suggests that inhibition may start as early as 100 ms after the sound onset. Additionally, the P3 response (typically starting around 200–250 ms; Wessel, 2018a) suggests that the effect may be observed even later in time. Therefore, while data from MEPs suggest transient inhibition, there is a relatively wide time window in which the effect could occur.

Evidence from eye movements also suggests variable time onsets for inhibition: some studies place it as early as 60 ms after sound onset (Vasilev et al., 2021), others around 80–100 ms (Graupner et al., 2007; Widmann et al., 2014) or 100–200 ms (Kadosh & Bonneh, 2022b; Valsecchi & Turatto, 2009), and yet others around 180 ms (Vasilev et al., 2019). The difference between sound onset and fixation onset appears to be a key variable in explaining its temporal dynamics (Vasilev et al., 2021). Therefore, there is a lot of uncertainty in how quickly novel sounds can affect eye movements. In this study, our main goal was to examine the timeline of novelty distraction in a more controlled environment.

One limitation of previous studies is that they have either used central stimuli presented at fixation (e.g., Kadosh & Bonneh, 2022b; Valsecchi & Turatto, 2009) or active vision tasks (e.g., reading, scene viewing). In both cases, the experimenter has no control over when participants make their next saccade or microsaccade. Therefore, while some of these tasks have greater ecological validity, they make it harder to determine the exact temporal onset of the effect. For this reason, the present study used simple saccade tasks that allow for greater temporal control in order to understand the time course of inhibition by novel sounds.

A secondary goal of the study was to examine whether unexpected sounds affect both voluntarily generated and more involuntarily generated saccades. Many human actions, such as driving, waving, and writing, are considered voluntary behaviors that are under conscious control. Conversely, involuntary behaviors, such as blinking, can arise in response to environmental factors. The pro−/anti‐saccade task (Hallett, 1978; Hallett & Adams, 1980; Munoz & Everling, 2004) is a highly controlled experimental paradigm that offers a unique opportunity to study the dichotomy between these two types of behavior in the context of eye movements, which represents one of the fastest motor responses in humans.

The pro‐saccade task involves the generation of a reflexive (somewhat involuntary) saccade toward a peripheral target location, facilitated by the oculomotor system's dominant feature of prioritizing sudden onset stimuli (Pratt & Trottier, 2005). The anti‐saccade task, on the contrary, involves the execution of a voluntary saccade in the mirror‐opposite direction of where the peripheral target appeared. Therefore, anti‐saccades require two separate actions: (1) inhibition of the reflexive response (pro‐saccade) to follow the peripheral stimulus; and (2) the translation of sensory‐motor plans to execute a voluntary saccade in the opposite direction (Munoz & Everling, 2004). As such, the anti‐saccade task is often considered as a measure of response inhibition that requires some amount of executive control. However, participants are not always successful in inhibiting the automatic response and make an error (i.e., execute a pro‐saccade instead of the required anti‐saccade) about 10%–30% of the time (Ettinger et al., 2003; Koval et al., 2004; Pierce & McDowell, 2016).

Interestingly, there is an overlap in the neural circuits involved in saccadic control in the pro−/anti‐saccade task and the purported fronto‐basal action‐stopping network that is activated by unexpected events (Wessel, 2018a; Wessel & Aron, 2013). In particular, performance on the pro−/anti‐saccade task also recruits frontal and parietal areas, as well as the basal ganglia (BG) (Coe & Munoz, 2017). This suggests that the activation of frontal areas and BG (particularly the STN) by unexpected sounds may lead to saccadic inhibition in these tasks due to them sharing common and interconnected brain networks.

### 1.1 Present study

We asked participants to perform the pro‐saccade and anti‐saccade task while listening to expected and unexpected sounds. We were interested in whether saccadic inhibition by unexpected sounds differs when performing the more reflective pro‐saccades versus the more voluntarily generated anti‐saccades. Critically, to test the time course of saccadic inhibition, we also manipulated the timing of the sound relative to the appearance of the saccade cue, starting from 150 ms before the cue to 25 ms after the cue.

Consistent with previous work (Graupner et al., 2007; Kadosh & Bonneh, 2022b; Valsecchi & Turatto, 2009; Vasilev et al., 2019, 2023; Widmann et al., 2014), we expected that novel sounds would lead to longer saccadic reaction times (SRTs) in both the pro‐saccade and anti‐saccade trials compared to standard sounds. Additionally, we expected that the inhibition would be transient and mostly occur when the sound is presented between 150 to 100 ms before the saccade cue. This is because the sound has been observed most commonly around that time in eye movements (e.g., Graupner et al., 2007; Kadosh & Bonneh, 2022b; Widmann et al., 2014). Furthermore, data from MEPs too suggests a similar onset (Iacullo et al., 2020; Wessel & Aron, 2013). Nevertheless, it is important to note that MEP studies have used different tasks and effector muscles, so the onset of inhibition in eye movements may not be identical.

In terms of differences between pro‐saccades and anti‐saccades, we expected greater inhibition by unexpected sounds in the anti‐saccade task because it involves greater recruitment of frontal areas compared to the pro‐saccade task (DeSouza & Everling, 2002; Furlan et al., 2016). Finally, because correct anti‐saccade performance requires inhibition of the reflexive pro‐saccade response, unexpected sounds may *improve* anti‐saccade accuracy if they are successful in inhibiting the oculomotor system. Therefore, novel sounds may lead to fewer anti‐saccade errors compared to standard sounds in the same way that they help behavioral stopping in the Go/No‐Go task (Wessel, 2017).

## 2 METHOD

The study had a 2 (*task*: pro‐saccade, anti‐saccade) × 2 (*sound*: standard, novel) × 8 (*sound onset*: −150, −125, −100, −75, −50, −25, 0, +25 ms relative to visual target onset) within‐subject design.

### 2.1 Participants

Seventy‐two members of the Bournemouth community (46 women)^2^ took part in return for 3.5 course credits or a £35 Amazon voucher. Their average age was 27.1 years (SD = 8.05 years; range: 18–49 years). Participants reported normal (or corrected‐to‐normal) vision, normal hearing, and no prior diagnosis of neurological disorders. All participants were naïve as to the purpose of the experiment and provided written informed consent. The study was approved by the Bournemouth University Research Ethics Committee (ID: 42319).

The sample size was determined a priori based on statistical simulations using the simR package (Green & Macleod, 2016) on previous data (Vasilev et al., 2021). With an *α* level of .05 and 30 novel sounds per condition per participant, the simulations suggested that about 50 participants were needed to achieve 95% power of detecting 75% of the expected effect size (*d* = 0.15). However, there is uncertainty in the true size of the novelty distraction effect (and its potential interactions) in new and previously untested tasks, as well as potential data loss (e.g., due to blinks). Therefore, we decided to be more conservative and set the sample size to 72 participants prior to data collection.

### 2.2 Apparatus

Participants' eye movements were recorded with the Eyelink 1000 system (SR Research, Ontario, Canada), which is a video‐based eye tracker with a sampling frequency of 1000 Hz. The average horizontal accuracy of the system was 0.25°–0.5°. While viewing was binocular, only the right eye was recorded.^3^ Participants' heads were stabilized with a forehead‐and‐chinrest. Testing was done in a small room illuminated by an overhead LED lamp. The visual stimuli were presented on a 24.5 inch Alienware 25 LCD monitor (resolution: 1920 × 1080; refresh rate: 244 Hz). The distance between the eye and the monitor was 62 cm. All sound stimuli were played on a Creative Sound Blaster Z soundcard (SB1500) at 65 ± 1.5 dB(A) through Bose QuietComfort 25 noise‐canceling headphones.

The experiment was programmed in Matlab 2021b (MathWorks, 2021) using the Psychophysics Toolbox v.3.15 (Brainard, 1997; Cornelissen et al., 2002; Pelli, 1997). The experiment was run on a Windows 10 PC (64 bit). The screen refresh latency was 8 ms and the sound output latency was 28 ms (measured with the Black Box ToolKit v2, Sheffield, UK). The software took these delays into account and ensured that the physical onset of the sound relative to the visual target onset corresponded exactly to the experimental sound onset condition (accuracy was measured to be within 1 ms, on average).

### 2.3 Stimuli

#### 2.3.1 Sounds

In 83.3% of all trials, participants heard the same (expected) “standard” sound. This was a 400 Hz sine wave tone that was 150 ms long with 10 ms fade‐in/ fade‐out ramps. In the remaining 16.7% of trials, participants heard an unexpected “novel” sound. The novel sounds were also 150 ms long and consisted of 240 unique environmental and nature sounds (e.g., a door closing, a bell, a siren, sea waves, city noises, as well as various animal sounds, such as a cat, dog, goat, birds, etc.). Ninety‐eight sounds were taken from Andrés et al. (2006) (originally from Escera et al., 1998); the remaining were created for this study (see https://osf.io/ph7te/). The novel sounds were presented in random order for each participant (once per task). All sounds were sampled at 44.1 kHz (stereo, 16‐bit).

#### 2.3.2 Pro‐saccade task

**FIGURE 1:**
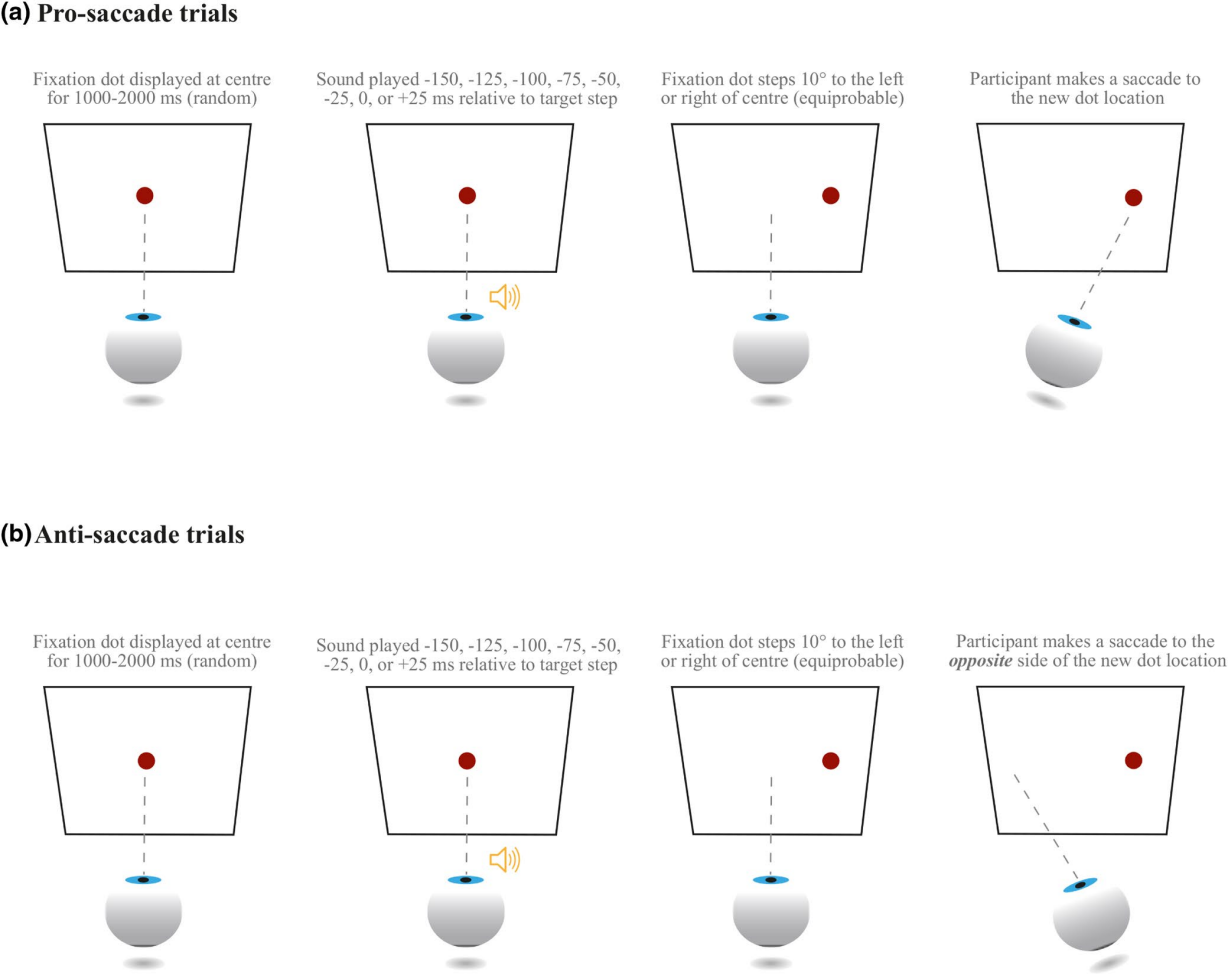
An illustration of the pro‐saccade (a) and anti‐saccade (b) tasks used in the experiment.

The two tasks, illustrated in Figure 1, followed Antoniades et al.'s (2013) standardized protocol. In the pro‐saccade task, participants made horizontal saccades to targets of 10° eccentricity relative to the center of the screen. An equal number of targets were presented to the left and to the right. The target was a circle with a diameter of 0.5°, colored dark red (RGB: 149, 0, 0) and presented against a white background. Trials started with a fixation dot (same as the target) at the center of the screen. The fixation dot remained on the screen for 1000–2000 ms (randomly drawn from a uniform distribution), after which it disappeared, and the target was presented either to the left or to the right for 1000 ms. The sound was presented in steps of 25 ms, starting from −150 ms before the target to +25 ms after the target appearance.

#### 2.3.3 Anti‐saccade task

The anti‐saccade task was identical to the pro‐saccade one, except that participants were instructed to execute a saccade in the *opposite* direction of the target step. For example, if the dot moved to the left of the screen, they were required to move their eyes to the right of the screen (and vice versa). Feedback was provided during the practice period and whenever participants made four consecutive mistakes in a row in the experimental blocks, to remind them of the instruction (Antoniades et al., 2013).

### 2.4 Procedure

Participants were tested in two sessions on two separate days (the average time between sessions was 5.15 days; SD = 7.08 days; range: 1–37 days). Each task was completed in a separate session block and their order was counter‐balanced across participants. We preferred a blocked design because interweaved trial presentation can affect response accuracy and the difference in SRTs between anti‐saccades and pro‐saccades (Zeligman & Zivotofsky, 2017). As such, the blocked presentation allowed us to maximize the differences between the two tasks in order to test how they are affected by unexpected sounds.

Within each task block, the trials were randomized with the constraint that no two novel sounds were played one after another and that the first five sounds and the last sound in the block were always standard ones. The randomization was done by grouping trials into sets of 48 (8 novel and 40 standard ones, corresponding to a single run of all conditions within a task). Participants completed 10 practice trials before the experimental ones. The experimental trials were then grouped into 10 smaller blocks of 144 trials each. Overall, in each experimental condition, participants completed 30 trials with a novel sound and 150 trials with a standard sound.

The task instructions were taken verbatim from Antoniades et al. (2013). In the pro‐saccade task, participants were instructed to look at the peripheral target as quickly as possible. In the anti‐saccade task, participants were instructed to look in the opposite direction of the target as quickly as possible. Note that the instructions did not emphasize making exact mirror saccades. Additionally, participants were told that they would hear different sounds but that they should try to ignore them and focus on the task.

Each testing session started with a 9‐point calibration and validation grid. Calibration accuracy was then monitored with a drift check every 15 trials and participants were re‐calibrated whenever necessary (but at least every 144 trials). Calibration accuracy of <0.5° was maintained throughout the experiment. There was a short break between each block and a longer break halfway through the session. Each session lasted about 1.5 to 1.75 h.

### 2.5 Data analysis

Saccades were detected with the Eyelink software algorithm. Samples were detected as saccades when they exceeded a velocity threshold of 35°/s and an acceleration threshold of 9500°/s. The dependent variables were: *saccadic reaction time* (the time difference between the onset of the peripheral target and the onset of the next saccade), *saccade amplitude* (the distance in visual angle that the eye traveled from the start of the saccade until the end of the saccade), and anti‐saccade *error rate* (proportion of trials where an incorrect response was executed).

Statistical analysis was done on the raw (unaggregated) data with (Generalized) Linear Mixed Models ((G)LLMs) using the lme4 package v.1.1–34 (Bates et al., 2015) in the R software v. 4.31 (R Core Team, 2024). Fixed effects were Sound Onset (−150, −125, −100, −75, −50, −25, 0, 25 ms), coded using successive differences contrast coding, and Sound (novel: 1, standard: −1) and Task (anti‐saccade: 1, pro‐saccade: −1), both coded using sum contrast coding. To test for more fine‐grained changes in the timeline of the effects, a post hoc analysis was also conducted where Sound Onset was treated as a continuous variable (including both linear and quadratic terms). The full results from these models are reported in the Supplementary Materials and we only highlight notable differences from the main model in the text.

Participants were included as a random intercept in the models (Baayen et al., 2008). Additionally, random slopes for Task, Sound, and Sound Onset were included (Barr et al., 2013). If the model failed to converge, the slopes were removed one by one until convergence was achieved. The formulas for each model were:

- SRT: lmer(log(SRT) ~ sound*task*onset +(task|sub), data = dat)
- Saccade amplitude: lmer(sacc_ampl ~ sound*task*onset +(sound|sub), data = dat)
- Error rate: glmer(error_rate ~ sound*onset + (1|sub), data = subset(dat, task == “ANTISACC”), family = binomial(link = “logit”)).

Saccadic reaction time was log‐transformed to improve the distribution of residuals. A Bonferroni correction was applied due to the use of 3 dependent variables. The results were considered statistically significant if the *p*‐value was ≤.016 (0.05/3). Significant interactions were followed up with Bonferroni‐adjusted *t*‐tests using the emmeans R package v.1.10.1 (Lenth, 2024). Empirical effect sizes are reported in Cohen's *d* (Cohen, 1988).

## 3 RESULTS

**FIGURE 2:**
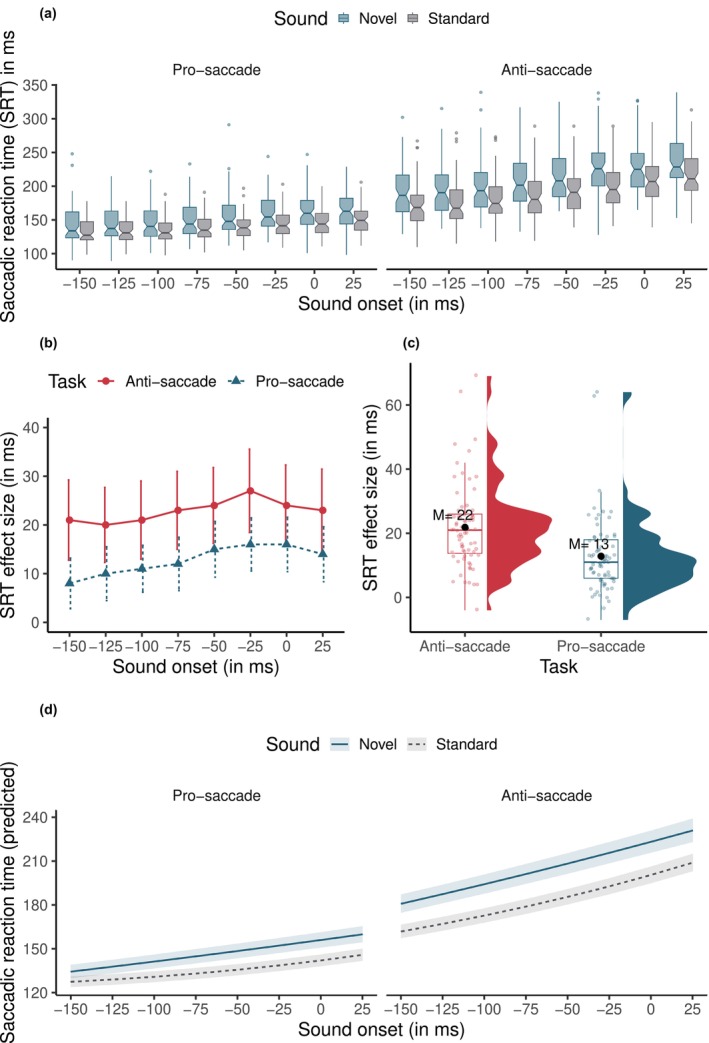
Saccadic reaction time data in the experiment. (a) boxplots show the data for each sound, onset condition, and task. Sound onsets are relative to the appearance of the target stimulus on the screen (at 0 ms). (b) shows the novelty distraction effect size (novel—standard) in saccadic reaction times for each sound onset condition. Error bars show ±1 SE. (c) shows the novelty distraction effect size for each task, aggregated over the onset conditions. Dots indicate the by‐participant means. (d) shows the predicted saccadic reaction times from a LMM model that treats sound onset as a continuous variable and includes both linear and quadratic terms for it. Shading indicates 95% CIs.

**FIGURE 3:**
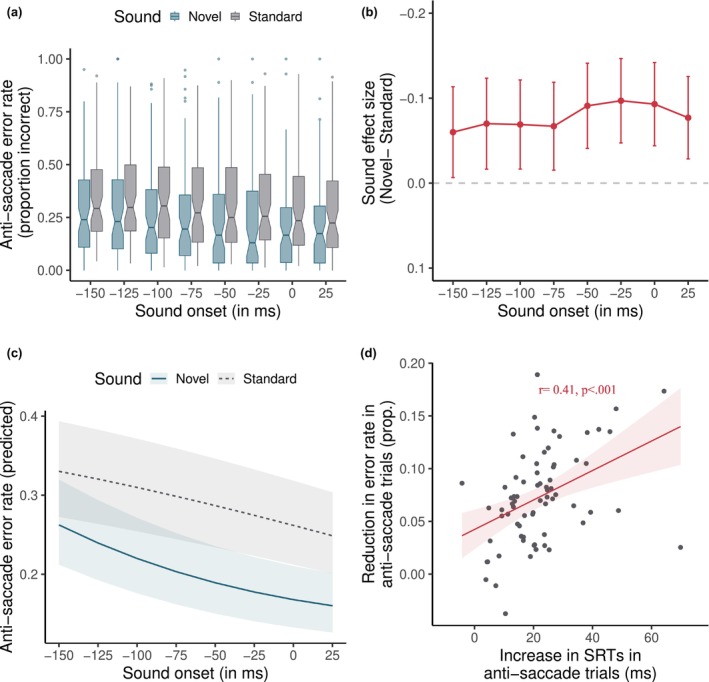
Anti‐saccade errors in the experiment. (a) shows boxplots of the error rate for each sound and delay condition. (b) shows the sound effect size for anti‐saccade error for each delay condition. Error bars show ±1 SE. (c) shows the predicted anti‐saccade error rate from a GLMM model that treats sound onset as a continuous variable and includes both linear and quadratic terms for it. Shading indicates 95% CIs. (d) shows a positive correlation between the reduction in error rate and the size of the novelty distraction effect in SRTs in the anti‐saccade task. In other words, participants with greater saccadic inhibition in SRTs also show a greater reduction in anti‐saccade errors. Dots show the individual effect size for each participant.

During data pre‐processing, trials with blinks were removed (*N* = 8152, 3.94%), as well as trials with saccade latencies smaller than 50 ms (*N* = 4961, 2.4%) or saccades with amplitude 2 SDs above/below the target eccentricity of 10° (*N* = 13,217, 6.39%) (Wenban‐Smith & Findlay, 1991). This left 87.4% of the data for analysis. Descriptive statistics are shown in Table 1 and the key results are visualized in Figures 2, 3.

**TABLE 1:** Mean saccadic reaction time, saccade amplitude, and error rate for each sound, onset, and task condition (SDs in parentheses).

Task	Sound	Sound onset	Saccadic reaction time (ms)	Saccade amplitude (°)	Error rate (prop. incorrect)
Pro‐saccade	Novel	−150 ms	140 (46)	9.98 (1.52)	0.01 (0.08)
Pro‐saccade	Standard	−150 ms	132 (42)	9.93 (1.67)	0.01 (0.08)
Pro‐saccade	Novel	−125 ms	144 (52)	10.10 (1.41)	<0.01 (0.03)
Pro‐saccade	Standard	−125 ms	134 (42)	9.95 (1.64)	0.01 (0.08)
Pro‐saccade	Novel	−100 ms	145 (43)	10.10 (1.40)	<0.01 (0.06)
Pro‐saccade	Standard	−100 ms	134 (39)	9.97 (1.58)	0.01 (0.08)
Pro‐saccade	Novel	−75 ms	150 (48)	10.20 (1.43)	<0.01 (0.04)
Pro‐saccade	Standard	−75 ms	138 (45)	9.97 (1.64)	0.01 (0.07)
Pro‐saccade	Novel	−50 ms	155 (54)	10.10 (1.44)	<0.01 (0.04)
Pro‐saccade	Standard	−50 ms	140 (43)	9.96 (1.60)	<0.01 (0.07)
Pro‐saccade	Novel	−25 ms	159 (51)	10.10 (1.58)	<0.01 (0.06)
Pro‐saccade	Standard	−25 ms	143 (42)	9.92 (1.64)	0.01 (0.08)
Pro‐saccade	Novel	0 ms	163 (51)	10.10 (1.37)	<0.01 (0.02)
Pro‐saccade	Standard	0 ms	147 (43)	9.91 (1.63)	0.01 (0.07)
Pro‐saccade	Novel	25 ms	165 (50)	10.10 (1.52)	<0.01 (0.06)
Pro‐saccade	Standard	25 ms	151 (46)	9.85 (1.73)	0.01 (0.08)
Anti‐saccade	Novel	−150 ms	195 (75)	9.23 (4.14)	0.26 (0.44)
Anti‐saccade	Standard	−150 ms	174 (65)	9.23 (4.15)	0.32 (0.47)
Anti‐saccade	Novel	−125 ms	197 (66)	9.12 (4.17)	0.26 (0.44)
Anti‐saccade	Standard	−125 ms	177 (64)	9.23 (4.15)	0.33 (0.47)
Anti‐saccade	Novel	−100 ms	203 (71)	9.11 (4.24)	0.24 (0.43)
Anti‐saccade	Standard	−100 ms	182 (65)	9.27 (4.18)	0.31 (0.46)
Anti‐saccade	Novel	−75 ms	211 (71)	9.30 (4.27)	0.23 (0.42)
Anti‐saccade	Standard	−75 ms	188 (65)	9.28 (4.19)	0.30 (0.46)
Anti‐saccade	Novel	−50 ms	220 (68)	9.34 (4.27)	0.20 (0.40)
Anti‐saccade	Standard	−50 ms	196 (65)	9.20 (4.20)	0.29 (0.45)
Anti‐saccade	Novel	−25 ms	230 (78)	9.21 (4.28)	0.19 (0.39)
Anti‐saccade	Standard	−25 ms	203 (67)	9.22 (4.21)	0.29 (0.45)
Anti‐saccade	Novel	0 ms	234 (72)	9.21 (4.28)	0.18 (0.38)
Anti‐saccade	Standard	0 ms	210 (69)	9.13 (4.20)	0.27 (0.45)
Anti‐saccade	Novel	25 ms	242 (74)	9.14 (4.19)	0.18 (0.39)
Anti‐saccade	Standard	25 ms	219 (70)	9.02 (4.26)	0.26 (0.44)

### 3.1 Saccadic reaction time (SRT)

The statistical results are shown in Table 2. We found a significant main effect of Sound (*b* = 0.048, SE = 0.003, *t* = 16.506, *p* < .001), indicating that novel sounds (*M* = 182 ms; SD = 70 ms) led to longer SRTs compared to standard sounds (*M* = 165 ms; SD = 62 ms), *d =* 0.25. Additionally, the results showed a significant main effect of Task (*b* = 0.152, SE = 0.008, *t* = 18.717, *p* < .001), with the anti‐saccade task (*M* = 197 ms; SD = 69 ms) leading to longer SRTs compared to the pro‐saccade task (*M* = 142 ms; SD = 45 ms), *d* = 0.90. This is in line with previous research (Hallett, 1978). Furthermore, the difference in each sound onset level to the next one was significant (all *p*‐values < .001; see Table 2 for the full results). As Figure 2a shows, SRTs increased slightly, in a linear fashion, as the sound was played closer in time to the onset of the saccade target.

**TABLE 2:** Linear mixed effect results for saccadic reaction time and saccade amplitude.

Predictors	Log (saccadic reaction time)				Saccade amplitude			
	Estimate	Std. error	*t* statistic	*p*	Estimate	Std. error	*t* statistic	*p*
(Intercept)	5.102	0.016	318.483	**<.001**	9.650	0.156	62.023	**<.001**
sound	0.048	0.003	16.506	**<.001**	0.045	0.017	2.688	.**007**
task	0.152	0.008	18.717	**<.001**	−0.352	0.009	−39.09	**<.001**
onset −150 vs. −125	0.020	0.003	6.573	**<.001**	−0.003	0.035	−0.072	.943
onset −125 vs. −100	0.021	0.003	6.758	**<.001**	0.025	0.035	0.703	.482
onset −100 vs. −75	0.033	0.003	10.768	**<.001**	0.048	0.035	1.357	.175
onset −75 vs. −50	0.035	0.003	11.218	**<.001**	−0.027	0.036	−0.772	.440
onset −50 vs. −25	0.033	0.003	10.529	**<.001**	−0.026	0.036	−0.715	.475
onset −25 vs. 0	0.022	0.003	7.151	**<.001**	−0.033	0.036	−0.920	.357
onset 0 vs. 25	0.029	0.003	9.256	**<.001**	−0.063	0.036	−1.759	.079
sound × task	0.007	0.001	9.214	**<.001**	−0.034	0.009	−3.818	**<.001**
sound × onset −150 vs. −125	0.002	0.003	0.588	.557	−0.012	0.035	−0.334	.739
sound × onset −125 vs. −100	0.001	0.003	0.478	.633	−0.000	0.035	−0.001	.999
sound × onset −100 vs. −75	0.003	0.003	0.918	.358	0.045	0.035	1.272	.203
sound × onset −75 vs. −50	0.003	0.003	1.103	.270	0.018	0.036	0.509	.611
sound × onset −50 vs. −25	0.002	0.003	0.729	.466	−0.010	0.036	−0.286	.775
sound × onset −25 vs. 0	−0.005	0.003	−1.666	.096	0.019	0.036	0.523	.601
sound × onset 0 vs. 25	−0.003	0.003	−1.050	.294	0.017	0.036	0.466	.641
task × onset −150 vs. −125	0.004	0.003	1.199	.231	−0.063	0.035	−1.791	.073
task × onset −125 vs. −100	0.009	0.003	2.768	.**006**	−0.011	0.035	−0.320	.749
task × onset −100 vs. −75	0.008	0.003	2.428	.**015**	0.040	0.035	1.135	.256
task × onset −75 vs. −50	0.009	0.003	3.010	.**003**	0.016	0.036	0.438	.661
task × onset −50 vs. −25	0.006	0.003	2.057	.040	−0.030	0.036	−0.852	.394
task × onset −25 vs. 0	0.002	0.003	0.502	.615	−0.014	0.036	−0.394	.693
task × onset 0 vs. 25	0.009	0.003	2.744	.**006**	−0.035	0.036	−0.963	.335
sound × task × onset −150 vs. −125	−0.004	0.003	−1.257	.209	−0.039	0.035	−1.103	.270
sound × task × onset −125 vs. −100	−0.005	0.003	−1.461	.144	−0.021	0.035	−0.584	.559
sound × task × onset −100 vs. −75	0.002	0.003	0.557	.578	0.033	0.035	0.933	.351
sound × task × onset −75 vs. −50	−0.003	0.003	−0.864	.388	0.048	0.036	1.346	.178
sound × task × onset −50 vs. −25	0.000	0.003	0.088	.930	−0.055	0.036	−1.538	.124
sound × task × onset −25 vs. 0	−0.004	0.003	−1.427	.154	0.027	0.036	0.750	.453
sound × task × onset 0 vs. 25	−0.001	0.003	−0.374	.708	−0.009	0.036	−0.257	.797
*Random effects*								
σ^2^	0.061					7.987		
τ_00_	0.018_sub_					1.737_sub_		
τ_11_	0.001_sub.sound_					0.014_sub.sound_		
	0.005_sub.task_							
ρ_01_	0.450					0.039_sub_		
	0.253							
ICC	0.260					0.179		
*N*	72_sub_					72_sub_		
Observations	180,818					180,818		
Marginal *R* ^2^/conditional *R* ^2^	0.247/0.443					.012/.189		

*Note*: Statistically significant *p*‐values are formatted in bold. A Bonferroni correction was applied and the significance threshold was 0.05/3 = 0.016.

Crucially, we found a significant Sound by Task interaction (*b* = 0.007, SE = 0.001, *t* = 9.214, *p* < .001). As Figure 2c shows, novel sounds were more distracting in the anti‐saccade task (*d* = 0.31) than in the pro‐saccade task (*d* = 0.26), *b* = 0.029, SE = 0.00315, *z* = 9.21, *p* < .001. Finally, the results showed a significant Task by Sound Onset interaction in a few of the sound onset windows. Specifically, the difference in SRTs between the anti‐saccade and pro‐saccade task increased from −125 to −100 ms (*b* = 0.009, SE = 0.003, *t* = 2.768, *p* = .006), from −100 to −75 (*b* = 0.008, SE = 0.003, *t* = 2.428, *p* = .015), from −75 to −50 (*b* = 0.009, SE = 0.003, *t* = 3.010, *p* = .003), and from 0 to +25 ms (*b* = 0.009, SE = 0.003, *t* = 2.744, *p* = .006).

When Sound Onset was treated as a continuous predictor, it exhibited a significant interaction with Sound (linear term: *b* = 0.969, SE = 0.343, *t* = 2.823, *p* = .005; quadratic term: *b* = −1.067, SE = 0.343, *t* = −3.108, *p* = .002; see Supplementary Materials for full details). As Figure 2d shows, the difference between novel and standard sounds increased slightly as the sound was played closer in time to the target, before reaching a plateau around −50 ms. There was also a significant three‐way interaction between Sound, Task, and Sound Onset in the linear (*b* = −1.724, SE = 0.343, *t* = −5.023, *p* < .001), but not the quadratic term (*b* = 0.165, SE = 0.343, *t* = 0.480, *p* = .631) for Sound Onset. As Figure 2d illustrates, there was a trend for the novelty distraction effect to be weaker in the pro‐saccade task compared to the anti‐saccade task and increase more strongly with Sound Onset.

To summarize, novel sounds led to longer SRTs compared to standard sounds and this effect was stronger in the anti‐saccade task compared to the pro‐saccade task. However, the novelty distraction effect was present across all sound onset delay conditions. It started a little weaker with sound onsets between −150 ms and −100 ms and then increased until reaching a plateau around −50 ms—a trend that was more apparent in the pro‐saccade task than the anti‐saccade task (see Figure 2b). Therefore, novel sounds led to a general increase in SRTs across all time intervals and there was only a small gradual increase with greater sound onsets.

Furthermore, a post hoc analysis (see Figure S1 in the Supplementary Materials) showed that the novelty distraction effect decreased, and then plateaued, as the experiment progressed, but generally remained significant by the end of the experiment. The only exception to this were the −150, −125, and − 75 ms sound onset conditions in the pro‐saccade task, where the effect of the novel sound was no longer significant toward the end of the experiment. This suggests that the inhibition in the first few time intervals (−150 to −75 ms) was smaller and/or wore off more quickly as participants habituated to the unexpected sounds.

### 3.2 Saccade amplitude

The statistical results are shown in Table 2. Saccade amplitudes, measured in degrees of visual angle, were significantly longer in the novel (*M* = 9.66; SD = 3.05) compared to the standard sound condition (*M* = 9.58; SD = 3.06), *b* = 0.045, SE = 0.017, *t* = 2.688, *p* = .007, though the effect size was marginal (*d* = 0.03). Additionally, saccade amplitudes were significantly longer in the pro‐saccade (*M* = 9.97; SD = 1.57) compared to the anti‐saccade task (*M* = 9.17; SD = 4.10), *b* = −0.352, SE = 0.009, *t* = −39.09, *p* < .001, *d* = −0.25. With the target consistently occurring at a 10° eccentricity, the data suggest that while mean saccade accuracy was very high for pro‐saccades (Nuthmann et al., 2016), it was considerably reduced for anti‐saccades, in line with previous research (Krappmann, 1998).

The interaction between Sound and Task was also significant (*b* = −0.034, SE = 0.009, *t* = −3.818, *p* < .001): the sound effect was present in the pro‐saccade task (*b* = 0.158, SE = 0.037, *t* = 4.247, *p* < .001, *d* = 0.09), but not in the anti‐saccade task (*b* = 0.021, SE = 0.038, *t* = 0.537, *p* = 1, *d* = 0). One possible explanation for this finding is that the more frequent standard sounds may have induced adaptation or repetition enhancement with successive saccades (Kadosh & Bonneh, 2022a), leading to slightly shorter saccades compared to novel sounds. Finally, the Supplementary model treating Sound Onset as a continuous predictor showed that the difference between novel and standard sounds in saccade amplitudes increased with greater sound onsets (see the Supplementary Materials for more details).

### 3.3 Anti‐saccade error rate

The statistical results are shown in Table 3. We found a main effect of Sound. As Figure 3a,b show, novel sounds (*M* = 0.22; SD = 0.41) led to a significantly lower proportion of errors in the anti‐saccade task compared to the standard sound (*M* = 0.30; SD = 0.46), *b* = −0.245, SE = 0.012, *z* = −19.998, *p* < .001, *d* = −0.16. Additionally, the error rate was significantly lower in the −50 ms (*M* = 0.27; SD = 0.45) compared to the −75 ms (*M* = 0.29; SD = 0.45) sound onset condition (*b* = −0.133, SE = 0.049, *z* = −2.728, *p* = .006), though the effect size was marginal (*d* = −0.03). There were no other significant differences.

**TABLE 3:** Generalized linear mixed effect results for anti‐saccade error rate.

	Anti‐saccade error rate			
Predictors	Estimate	Std. error	*z* statistic	*p*
(Intercept)	−1.137	0.139	−8.159	**<.001**
sound	−0.245	0.012	−19.998	**<.001**
onset −150 vs. −125	0.014	0.046	0.306	.759
onset −125 vs. −100	−0.107	0.046	−2.310	.021
onset −100 vs. −75	−0.095	0.047	−2.009	.045
onset −75 vs. −50	−0.133	0.049	−2.728	.**006**
onset −50 vs. −25	−0.030	0.050	−0.606	.544
onset −25 vs. 0	−0.057	0.051	−1.121	.262
onset 0 vs. 25	−0.035	0.052	−0.671	.502
sound × onset −150 vs. −125	−0.028	0.046	−0.612	.541
sound × onset −125 vs. −100	0.014	0.046	0.295	.768
sound × onset −100 vs. −75	−0.021	0.047	−0.456	.649
sound × onset −75 vs. −50	−0.090	0.049	−1.856	.063
sound × onset −50 vs. −25	−0.015	0.050	−0.290	.772
sound × onset −25 vs. 0	0.018	0.051	0.356	.722
sound × onset 0 vs. 25	0.065	0.052	1.240	.215
*Random effects*				
σ^2^	3.290			
τ_00sub_	1.444			
ICC	0.305			
*N*_sub_	72			
Observations	85,699			
Marginal *R* ^2^/conditional *R* ^2^	.012/.313			

*Note*: Statistically significant *p*‐values are formatted in bold. A Bonferroni correction was applied and the significance threshold was 0.05/3 = 0.016.

In the Supplementary model treating Sound Onset as a continuous predictor, there was a significant interaction between Sound and Sound Onset in both the linear (*b* = −10.754, SE = 0.890, *z* = −12.086, *p* < .001) and quadratic terms (*b* = 4.985, SE = 1.031, *z* = 4.835, *p* < .001). As Figure 3c shows, the difference in error rates between novel and standard sounds increased with Sound Onset, until reaching a plateau around −50 ms. This mirrors the pattern in SRTs and suggests that the inhibition of incorrect responses was smallest in the earlier Sound Onset conditions.

Therefore, the unexpected novel sounds generally aided response inhibition in the anti‐saccade task – they helped subjects suppress the execution of erroneous pro‐saccades, thereby increasing the number of correct responses. Additionally, as Figure 3d shows, the reduction in error rate in the anti‐saccade task was moderately correlated with the increase in SRTs by novel sounds. In other words, participants who showed greater oculomotor inhibition in SRTs in the anti‐saccade task were also more likely to have a lower error rate.

**FIGURE 4:**
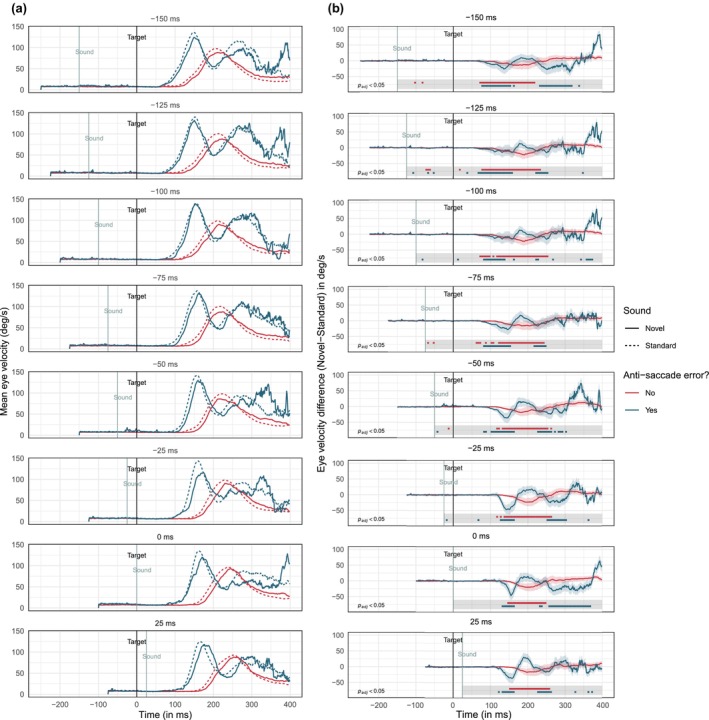
Mean eye velocity in the anti‐saccade task between novel and standard sounds across all sound onset conditions. Separate lines are plotted for trials on which participants made an anti‐saccade error (i.e., executed a pro‐saccade) and trials on which they made the correct response. (a) shows the velocity profiles for standard sounds (dashed lines) and novel sounds (solid lines) on correct/incorrect trials (see the main text for more information). (b) shows the difference between the standard and novel curves from (a). Negative differences indicate slower eye velocity following novel sounds. Eye velocities were extracted from 100 ms before the sound onset to 400 ms following the visual target onset (velocities after 400 ms are not included as there were too few samples for reliable estimation across the different cells). The bottom of each plot shows the time intervals (solid horizontal lines) during which the decrease in velocity was statistically significant. This was estimated using 5 ms bins and applying a Bonferroni correction to all *p*‐values.

To test for more subtle evidence of inhibition in anti‐saccades, we performed a post hoc analysis of the eye velocity for trials that contained an error and trials that did not contain an error. The velocities averaged across trials and subjects (see Figure 4a) showed the typical acceleration/ deacceleration curve around 150–350 ms for correct anti‐saccade trials. Incorrect trials showed an average velocity curve that occurred earlier (75–250 ms), replicating the typical finding that errors are executed faster than correct responses (Coe & Munoz, 2017). There were also bimodal/trimodal velocity peaks afterward, due to participants performing a corrective saccade to the correct area of the screen on some trials (~ 14% of the time). Of interest, the difference between standard sounds (dashed line) and novel sounds (solid line) showed that eye velocity was generally lower following novel sounds, particularly during the start of the saccade.

A closer examination of the difference between the two velocity curves (see Figure 4b) suggested that novel sounds led to a more sustained reduction in eye velocity on correct trials compared to incorrect trials. This was especially the case in the critical time window of about 100–250 ms when most anti‐saccades would have been executed. Therefore, while these results need to be interpreted with caution, they suggest that, on correct trials, novel sounds were more successful at inhibiting eye velocities. Whether this is related to the improvement in anti‐saccade performance remains to be tested.

## 4 DISCUSSION

The present study tested how quickly novel sounds begin to affect eye‐movement responses and whether this differs between voluntary anti‐saccades and reflexive pro‐saccades. For both types of saccades, novel sounds led to significantly longer SRTs compared to standard sounds. This finding is consistent with previous work using reading and scene viewing as well as fixation tasks (Graupner et al., 2007; Kadosh & Bonneh, 2022b; Valsecchi & Turatto, 2009; Vasilev et al., 2019, 2021, 2023; Widmann et al., 2014). Thus, unexpected sounds appear to have a general inhibitory effect on eye‐movement responses, supporting the notion that unexpected events cause global inhibition of motor actions (Wessel & Aron, 2013, 2017).

The present study builds upon previous work on microsaccades (Kadosh & Bonneh, 2022b; Valsecchi & Turatto, 2009; Widmann et al., 2014) by showing that oculomotor inhibition also extends to larger saccadic eye movements, such as pro‐saccades and anti‐saccades. Interestingly, the inhibition effect of novel sounds was stronger (albeit also more variable) in anti‐saccades compared to pro‐saccades, suggesting that voluntarily generated saccades are inhibited to a greater extent. Despite the increase in SRTs, novel sounds had a limited effect on saccade amplitudes, confirming previous reports that they don't inhibit saccade execution (Vasilev et al., 2021). This result was further confirmed by analyzing saccade velocities as a function of the time between playing the sound and the saccade onset (see the Supplementary Materials for more information).

The key manipulation of sound onset timing revealed an interesting, if surprising, pattern of results. The novelty distraction effect was present in all time intervals. The effect was weaker in the earlier sound onset conditions (between −150 and −100 ms), particularly in the pro‐saccade task, and gradually increased until reaching a plateau around −50 ms before the saccade target. Therefore, contrary to expectations, the effect was not transient for the time intervals that were tested. Rather, novel sounds led to a general inhibition of eye‐movement responses across all intervals. Even so, there was some evidence that the effect in the pro‐saccade task was weaker and started to disappear toward the end of the experiment when the sound was presented from 150 to 75 ms before the saccade target (see the Supplemental Materials). This further suggests that some of the earlier onset conditions led to weaker inhibition in the pro‐saccade task. Because we did not include sound onsets before −150 ms or after 25 ms, we cannot clearly state when the inhibition starts or stops. However, our data shows that the oculomotor system is inhibited during a window of 175 ms that was identified as most plausible based on previous evidence.

These results may appear to contradict data from MEPs, which suggests that global motor inhibition occurs at 150 ms following the sound onset but already disappears 25 ms later (Iacullo et al., 2020; Wessel & Aron, 2013). However, the present study only measured motor output latency in task‐relevant muscles (controlling the eye), whereas previous studies have mostly examined excitability in task‐irrelevant muscles. MEPs are thought to measure cortico‐spinal tract excitability following TMS stimulation (Duque et al., 2017) and usually occur within 10–50 ms of the TMS pulse (Rossini et al., 2015; Wilson et al., 2021). Therefore, they only give a small window of information about the excitability of the nervous system. SRTs, in contrast, typically have a latency of 100–250 ms (Edelman et al., 2006; Fischer & Weber, 1992) and reflect the more variable nature of eye‐movement programming. Therefore, both methods provide different information about motor inhibition and their timelines may not match up precisely. Additionally, the time course for global motor inhibition may be more flexible, with some studies suggesting it can start as early as 100 ms after sound onset (Novembre et al., 2018; Tatz et al., 2023). In this sense, the present data only suggests that the planning of task‐relevant eye movements is disrupted within the 175 ms window that we tested. However, it does not tell us how task‐irrelevant muscle activity may be affected.

This pattern of results may not be surprising given that previous eye‐movement studies have found the effect at different times, approximately along the time window that we tested (Graupner et al., 2007; Kadosh & Bonneh, 2022b; Vasilev et al., 2019, 2021; Widmann et al., 2014). However, it is worth noting that saccadic inhibition by visual distractors typically requires a close temporal overlap between the processing of the distractor and saccade programming (e.g., Bompas & Sumner, 2009; Reingold & Stampe, 2004). This suggests that unexpected sounds may have a broader inhibitory effect by influencing a sufficient proportion of SRTs at each time window. It is also interesting that the inhibition was slightly stronger than previous studies utilizing more “natural” tasks such as reading (Vasilev et al., 2019, 2021, 2023). We speculate that this may be because the sounds overlap more strongly with the saccadic programming stages, which is harder to achieve in active vision tasks where the decision of when to make an eye movement is also affected by other cognitive and perceptual processes. Additionally, it is possible that the use of novel sounds (as compared to pitch deviants) creates greater acoustical deviance, which further increases oculomotor inhibition (Kadosh & Bonneh, 2022a).

Interestingly, novel sounds also *reduced* errors in the anti‐saccade task across all sound onset intervals, suggesting that they aided inhibitory control. This provides further evidence that the effect of novel sounds is truly inhibitory in nature. While this result may appear counter‐intuitive at first, it is important to keep in mind that correct performance in the anti‐saccade task requires two separate processes: (1) suppressing the execution of the reflexive response (pro‐saccade) and (2) voluntary programming of a saccade in the opposite direction (anti‐saccade). It is only the first process that is captured by the anti‐saccade error rate.

Indeed, the present data indicates that novel sounds helped suppress this reflexive, stimulus‐driven behavior when it is not required by the task, in favor of more voluntary‐driven behavior. Suppression of pro‐saccades in the anti‐saccade task depends on top‐down inhibition of neurons in areas such as the frontal eye fields and the intermediate layer of the superior colliculus (Everling et al., 1998; Everling & Munoz, 2000; Munoz & Everling, 2004). We speculate that the activation of the fronto‐basal action‐stopping network (Diesburg & Wessel, 2021; Wessel & Aron, 2013) by unexpected sounds may facilitate this inhibition, both through the activation of frontal areas and the STN.

The STN is thought to play a key role in mediating the global motor inhibition response and receives direct cortical projections via the hyperdirect pathway (Diesburg & Wessel, 2021; Wessel & Aron, 2013). The STN also receives similar projections from key areas involved in saccadic control (such as the frontal eye fields; Nambu et al., 2002) and can enhance the inhibition of superior colliculus neurons, thus suppressing the generation of saccades (Bakhtiari et al., 2020; Hikosaka et al., 2000; Watanabe & Munoz, 2011). Such inhibition may be stronger in the anti‐saccade task as it involves greater frontal/parietal activation than the pro‐saccade task (DeSouza & Everling, 2002; Furlan et al., 2016). Additionally, patients with frontal/ BG disorders show a distinct reduction in anti‐saccade accuracy (e.g., Amador et al., 2006; Goto et al., 2010; Guitton et al., 1985; Ouerfelli‐Ethier et al., 2018), thus underscoring the importance of these areas for task performance.

Not many factors are known to reduce anti‐saccade errors, especially in healthy adults. Some studies suggest that practice on the task (Dyckman & McDowell, 2005; Montenegro & Edelman, 2019), as well as certain chemicals such as nicotine (Larrison et al., 2004; Petrovsky et al., 2013) and antipsychotic drugs (Burke & Reveley, 2002), which potentially affect pre‐frontal cortex neurons, lead to reduced errors. However, some attentional influences have been noted as well. For example, the presentation of an auditory or visual cue at the target location (Karatekin, 2006) and dual‐task paradigms where participants also have to judge luminance changes (Evens & Ludwig, 2010; but cf. Kristjánsson et al., 2001) both lead to a reduction in anti‐saccade errors. These latter results suggest that increased task demands and the cuing of attention may modulate successful anti‐saccade performance. In fact, novel sounds may have a similar effect by briefly diverting attention away from the target, which could potentially make the execution of the erroneous pro‐saccade less likely in the first place. This would be consistent with Wessel's (2018a) proposal that the motor inhibition and attention‐orienting responses are part of the same cascade and may be inseparable from each other.

In the Pause‐then‐Cancel model (Diesburg & Wessel, 2021), a Pause process is generated every time an unexpected event occurs, resulting in global motor inhibition and the orientation of attention. A Cancel process is then also programmed in parallel, which aims to readjust (or completely cancel) motor programs via activation of the pre‐SMA (Diesburg & Wessel, 2021). While we cannot distinguish between these two processes, it is possible that both may be at play here. The Pause process may be responsible for the general inhibition of oculomotor plans, which manifests itself as slower SRTs. On the contrary, the Cancel process could also affect saccadic programming, for example, by reinstating the oculomotor plans that were put on hold or by canceling those that are task‐inappropriate (such as executing a pro‐saccade instead of the required anti‐saccade). Future neuroimaging work could potentially shed light on this, for example by looking for pre‐SMA activation as evidence for the Cancel process.

It may be surprising that unexpected sounds affected *both* error rates and SRTs in the anti‐saccade task. However, the two effects are complementary to each other, as they both indicate that the oculomotor system was inhibited. In fact, as Figure 3d shows, the two effects are moderately correlated and share some variance. Further research is needed to understand if they are both caused by similar processes. However, it is worth noting that Dutra et al. (2018) also observed a similar correlation between inhibition of MEPs and successful stopping in the Go/No‐Go task‐ suggesting that the degree of inhibition is related to actual behavioral stopping. While in the present task, the inhibition in SRTs is, to some extent, confounded with the behavioral outcome, future studies could try to disentangle these.

## 5 CONCLUSION

Previous research has established that unexpected sounds yield inhibition of microsaccades (Kadosh & Bonneh, 2022b; Valsecchi & Turatto, 2009; Widmann et al., 2014) and saccadic eye movements during reading and scene viewing (Graupner et al., 2007; Rettie et al., 2024; Vasilev et al., 2019, 2021, 2023). The present study showed that unexpected sounds have a general inhibitory effect on reflexive pro‐saccade responses and the more voluntary anti‐saccade responses. This inhibition was found to emerge quickly and to be relatively constant for sounds presented between 150 ms before the target to 25 ms after the target. However, the effect was weaker for sounds presented between 150 ms to 100 ms before the target, particularly for pro‐saccades. Additionally, unexpected sounds reduced error rates on the anti‐saccade task, suggesting that they aided inhibitory control and helped participants reduce their reliance on reflexive, stimulus‐driven behavior. These results raise the possibility that unexpected events may exert a global suppressive effect on the oculomotor system.

## AUTHOR CONTRIBUTIONS

**Martin R. Vasilev:** Conceptualization; data curation; formal analysis; funding acquisition; investigation; methodology; project administration; resources; software; supervision; validation; visualization; writing – original draft; writing – review and editing. **Zeynep G. Ozkan:** Data curation; formal analysis; investigation; software; visualization; writing – original draft. **Julie A. Kirkby:** Conceptualization; writing – original draft; writing – review and editing. **Antje Nuthmann:** Conceptualization; visualization; writing – original draft; writing – review and editing. **Fabrice B. R. Parmentier:** Conceptualization; funding acquisition; methodology; resources; writing – original draft; writing – review and editing.

## FUNDING INFORMATION

This work was funded by a small grant from the Experimental Psychology Society (UK) awarded to M.R.V, as well as a research grant PID2020‐114117GB‐I00 awarded to F.B.R.P. funded by MICIU/AEI /10.13039/501100011033. It was conducted while M.R.V. was at Bournemouth University.

## Supporting information


**Data S1:** Modulation of novelty distraction by trial order.

## Data Availability

All data, analysis code, and materials from this study are available at: https://osf.io/9rb6n/.

## Footnotes

^1^
This effect has also been called “oculomotor freezing” (White & Rolfs, 2016) and “oculomotor inhibition” (Bonneh et al., 2016).

^2^
A further three participants dropped out after the first session and were replaced.

^3^
For 7 participants, the left eye was used instead due to tracking issues with glasses/contact lenses.

## References

[psyp14728-bib-0001] Amador, S. C. , Hood, A. J. , Schiess, M. C. , Izor, R. , & Sereno, A. B. (2006). Dissociating cognitive deficits involved in voluntary eye movement dysfunctions in Parkinson's disease patients. Neuropsychologia, 44(8), 1475–1482. 10.1016/j.neuropsychologia.2005.11.015 16376954

[psyp14728-bib-0002] Andrés, P. , Parmentier, F. B. R. , & Escera, C. (2006). The effect of age on involuntary capture of attention by irrelevant sounds: A test of the frontal hypothesis of aging. Neuropsychologia, 44(12), 2564–2568. 10.1016/j.neuropsychologia.2006.05.005 16797613

[psyp14728-bib-0003] Antoniades, C. , Ettinger, U. , Gaymard, B. , Gilchrist, I. , Kristjánsson, A. , Kennard, C. , John Leigh, R. , Noorani, I. , Pouget, P. , Smyrnis, N. , Tarnowski, A. , Zee, D. S. , & Carpenter, R. H. S. (2013). An internationally standardised antisaccade protocol. Vision Research, 84, 1–5. 10.1016/j.visres.2013.02.007 23474300

[psyp14728-bib-0004] Baayen, H. , Davidson, D. J. , & Bates, D. M. (2008). Mixed‐effects modeling with crossed random effects for subjects and items. Journal of Memory and Language, 59(4), 390–412. 10.1016/j.jml.2007.12.005

[psyp14728-bib-0005] Badry, R. , Mima, T. , Aso, T. , Nakatsuka, M. , Abe, M. , Fathi, D. , Foly, N. , Nagiub, H. , Nagamine, T. , & Fukuyama, H. (2009). Suppression of human cortico‐motoneuronal excitability during the stop‐signal task. Clinical Neurophysiology, 120(9), 1717–1723. 10.1016/j.clinph.2009.06.027 19683959

[psyp14728-bib-0006] Bakhtiari, S. , Altinkaya, A. , Pack, C. C. , & Sadikot, A. F. (2020). The role of the subthalamic nucleus in inhibitory control of oculomotor behavior in Parkinson's disease. Scientific Reports, 10(1), 1–11. 10.1038/s41598-020-61572-4 32214128 PMC7096507

[psyp14728-bib-0007] Barr, D. J. , Levy, R. , Scheepers, C. , & Tily, H. J. (2013). Random effects structure for confirmatory hypothesis testing: Keep it maximal. Journal of Memory and Language, 68(3), 255–278. 10.1016/j.jml.2012.11.001 PMC3881361 24403724

[psyp14728-bib-0008] Bates, D. , Mächler, M. , Bolker, B. , & Walker, S. (2015). Fitting linear mixed‐effects models using lme4. Journal of Statistical Software, 67(1), 1–48. 10.18637/jss.v067.i01

[psyp14728-bib-0009] Berti, S. (2012). Automatic processing of rare versus novel auditory stimuli reveal different mechanisms of auditory change detection. Neuroreport, 23(7), 441–446. 10.1097/WNR.0b013e32835308b5 22440977

[psyp14728-bib-0010] Bompas, A. , & Sumner, P. (2009). Temporal dynamics of saccadic distraction. Journal of Vision, 9(9), 1–14. 10.1167/9.9.17 19761350

[psyp14728-bib-0011] Bonneh, Y. S. , Adini, Y. , & Polat, U. (2016). Contrast sensitivity revealed by spontaneous eyeblinks: Evidence for a common mechanism of oculomotor inhibition. Journal of Vision, 16(7), 1–15. 10.1167/16.7.1 27135194

[psyp14728-bib-0012] Brainard, D. H. (1997). The psychophysics toolbox. Spatial Vision, 10(4), 433–436. 10.1163/156856897X00357 9176952

[psyp14728-bib-0013] Buonocore, A. , & Hafed, Z. M. (2023). The inevitability of visual interruption. Journal of Neurophysiology, 130(2), 225–237. 10.1152/jn.00441.2022 37377194

[psyp14728-bib-0014] Buonocore, A. , & McIntosh, R. D. (2012). Modulation of saccadic inhibition by distractor size and location. Vision Research, 69, 32–41. 10.1016/j.visres.2012.07.010 22842403

[psyp14728-bib-0015] Burke, J. G. , & Reveley, M. A. (2002). Improved antisaccade performance with risperidone in schizophrenia. Journal of Neurology, Neurosurgery & Psychiatry, 72(4), 449–454. 10.1136/JNNP.72.4.449 11909901 PMC1737823

[psyp14728-bib-0016] Cai, W. , Oldenkamp, C. L. , & Aron, A. R. (2012). Stopping speech suppresses the task‐irrelevant hand. Brain and Language, 120(3), 412–415. 10.1016/j.bandl.2011.11.006 22206872 PMC3533487

[psyp14728-bib-0017] Coe, B. C. , & Munoz, D. P. (2017). Mechanisms of saccade suppression revealed in the anti‐saccade task. Philosophical Transactions of the Royal Society, B: Biological Sciences, 372(1718), 1–10. 10.1098/rstb.2016.0192 PMC5332851 28242726

[psyp14728-bib-0018] Cohen, J. (1988). Statistical power analysis for the behavioral sciences (2nd ed.). Lawrence Erlbaum Associates.

[psyp14728-bib-0019] Cornelissen, F. W. , Peters, E. M. , & Palmer, J. (2002). The Eyelink toolbox: Eye tracking with MATLAB and the psychophysics toolbox. Behavior Research Methods, Instruments, & Computers, 34(4), 613–617. 10.3758/BF03195489 12564564

[psyp14728-bib-0020] Dalton, P. , & Hughes, R. W. (2014). Auditory attentional capture: Implicit and explicit approaches. Psychological Research, 78(3), 313–320. 10.1007/s00426-014-0557-5 24643575

[psyp14728-bib-0021] DeSouza, J. F. X. , & Everling, S. (2002). Neural correlates for preparatory set associated with pro‐saccades and anti‐saccades in humans investigated with event‐related fMRI. Journal of Vision, 2(7), 1016–1023. 10.1167/2.7.578 12574477

[psyp14728-bib-0022] Diesburg, D. A. , & Wessel, J. R. (2021). The pause‐then‐cancel model of human action‐stopping: Theoretical considerations and empirical evidence. Neuroscience and Biobehavioral Reviews, 129(April), 17–34. 10.1016/j.neubiorev.2021.07.019 34293402 PMC8574992

[psyp14728-bib-0023] Duque, J. , Greenhouse, I. , Labruna, L. , & Ivry, R. B. (2017). Physiological markers of motor inhibition during human behavior. Trends in Neurosciences, 40(4), 219–236. 10.1016/j.tins.2017.02.006 28341235 PMC5389740

[psyp14728-bib-0024] Dutra, I. C. , Waller, D. A. , & Wessel, J. R. (2018). Perceptual surprise improves action stopping by nonselectively suppressing motor activity via a neural mechanism for motor inhibition. Journal of Neuroscience, 38(6), 1482–1492. 10.1523/JNEUROSCI.3091-17.2017 29305533 PMC5815349

[psyp14728-bib-0025] Dyckman, K. A. , & McDowell, J. E. (2005). Behavioral plasticity of antisaccade performance following daily practice. Experimental Brain Research, 162(1), 63–69. 10.1007/s00221-004-2105-9 15551081

[psyp14728-bib-0026] Edelman, J. A. , Valenzuela, N. , & Barton, J. J. S. (2006). Antisaccade velocity, but not latency, results from a lack of saccade visual guidance. Vision Research, 46(8–9), 1411–1421. 10.1016/j.visres.2005.09.013 16260025

[psyp14728-bib-0027] Escera, C. , Alho, K. , Winkler, I. , & Näätänen, R. (1998). Neural mechanisms of involuntary attention to acoustic novelty and change. Journal of Cognitive Neuroscience, 10(5), 590–604. 10.1162/089892998562997 9802992

[psyp14728-bib-0028] Ettinger, U. , Kumari, V. , Crawford, T. J. , Davis, R. E. , Sharma, T. , & Corr, P. J. (2003). Reliability of smooth pursuit, fixation, and saccadic eye movements. Psychophysiology, 40(4), 620–628. 10.1111/1469-8986.00063 14570169

[psyp14728-bib-0029] Evens, D. R. , & Ludwig, C. J. H. (2010). Dual‐task costs and benefits in anti‐saccade performance. Experimental Brain Research, 205(4), 545–557. 10.1007/s00221-010-2393-1 20714711

[psyp14728-bib-0030] Everling, S. , Dorris, M. C. , & Munoz, D. P. (1998). Reflex suppression in the anti‐saccade task is dependent on prestimulus neural processes. Journal of Neurophysiology, 80(3), 1584–1589. 10.1152/jn.1998.80.3.1584 9744965

[psyp14728-bib-0031] Everling, S. , & Munoz, D. P. (2000). Neuronal correlates for preparatory set associated with pro‐saccades and anti‐saccades in the primate frontal eye field. Journal of Neuroscience, 20(1), 387–400. 10.1523/jneurosci.20-01-00387.2000 10627615 PMC6774131

[psyp14728-bib-0032] Fischer, B. , & Weber, H. (1992). Characteristics of “anti” saccades in man. Experimental Brain Research, 89(2), 415–424. 10.1007/BF00228257 1623983

[psyp14728-bib-0033] Furlan, M. , Smith, A. T. , & Walker, R. (2016). An fMRI investigation of preparatory set in the human cerebral cortex and superior colliculus for pro‐ and anti‐saccades. PLoS One, 11(7), e0158337. 10.1371/journal.pone.0158337 27391390 PMC4938211

[psyp14728-bib-0034] Goto, Y. , Hatakeyama, K. , Kitama, T. , Sato, Y. , Kanemura, H. , Aoyagi, K. , Sugita, K. , & Aihara, M. (2010). Saccade eye movements as a quantitative measure of frontostriatal network in children with ADHD. Brain and Development, 32(5), 347–355. 10.1016/j.braindev.2009.04.017 19505783

[psyp14728-bib-0035] Graupner, S. T. , Velichkovsky, B. M. , Pannasch, S. , & Marx, J. (2007). Surprise, surprise: Two distinct components in the visually evoked distractor effect. Psychophysiology, 44(2), 251–261. 10.1111/j.1469-8986.2007.00504.x 17343709

[psyp14728-bib-0036] Green, P. , & Macleod, C. J. (2016). SIMR: An R package for power analysis of generalized linear mixed models by simulation. Methods in Ecology and Evolution, 7(4), 493–498. 10.1111/2041-210X.12504

[psyp14728-bib-0037] Guitton, D. , Buchtel, H. A. , & Douglas, R. M. (1985). Frontal lobe lesions in man cause difficulties in suppressing reflexive glances and in generating goal‐directed saccades. Experimental Brain Research, 58(3), 455–472. 10.1007/BF00235863 4007089

[psyp14728-bib-0038] Hallett, P. E. (1978). Primary and secondary goals defined by instructions. Vision Research, 18(10), 1279–1296. 726270 10.1016/0042-6989(78)90218-3

[psyp14728-bib-0039] Hallett, P. E. , & Adams, B. D. (1980). The predictability of saccadic latency in a novel voluntary oculomotor task. Vision Research, 20(4), 329–339. 10.1016/0042-6989(80)90019-X 7414965

[psyp14728-bib-0040] Hikosaka, O. , Takikawa, Y. , & Kawagoe, R. (2000). Role of the basal ganglia in the control of purposive saccadic eye movements. Physiological Reviews, 80(3), 953–978. 10.1152/physrev.2000.80.3.953 10893428

[psyp14728-bib-0041] Horváth, J. , Winkler, I. , & Bendixen, A. (2008). Do N1/MMN, P3a, and RON form a strongly coupled chain reflecting the three stages of auditory distraction? Biological Psychology, 79(2), 139–147. 10.1016/j.biopsycho.2008.04.001 18468765

[psyp14728-bib-0042] Iacullo, C. , Diesburg, D. A. , & Wessel, J. R. (2020). Non‐selective inhibition of the motor system following unexpected and expected infrequent events. Experimental Brain Research, 238, 2701–2710. 10.1101/2020.03.25.008789 32948892 PMC7717674

[psyp14728-bib-0043] Kadosh, O. , & Bonneh, Y. S. (2022a). Fixation‐related saccadic inhibition in free viewing in response to stimulus saliency. Scientific Reports, 12(1), 1–12. 10.1038/s41598-022-10605-1 35459790 PMC9033846

[psyp14728-bib-0044] Kadosh, O. , & Bonneh, Y. S. (2022b). Involuntary oculomotor inhibition markers of saliency and deviance in response to auditory sequences. Journal of Vision, 22(5), 1–19. 10.1167/jov.22.5.8 PMC9055552 35475911

[psyp14728-bib-0093] Karatekin, C. (2006). Improving antisaccade performance in adolescents with attention‐deficit/hyperactivity disorder (ADHD). Experimental Brain Research, 174, 324–341. 10.1007/s00221-006-0467-x 16639499

[psyp14728-bib-0045] Koval, M. J. , Ford, K. A. , & Everling, S. (2004). Effect of stimulus probability on anti‐saccade error rates. Experimental Brain Research, 159(2), 268–272. 10.1007/s00221-004-2104-x 15549282

[psyp14728-bib-0046] Krappmann, P. (1998). Accuracy of visually and memory‐guided antisaccades in man. Vision Research, 38(19), 2979–2985. 10.1016/S0042-6989(98)00101-1 9797993

[psyp14728-bib-0047] Kristjánsson, Á. , Chen, Y. , & Nakayama, K. (2001). Less attention is more in the preparation of antisaccades, but not prosaccades. Nature Neuroscience, 4(10), 1037–1042. 10.1038/nn723 11547337

[psyp14728-bib-0048] Larrison, A. L. , Briand, K. A. , & Sereno, A. B. (2004). Nicotine improves antisaccade task performance without affecting prosaccades. Human Psychopharmacology: Clinical and Experimental, 19(6), 409–419. 10.1002/hup.604 15303245

[psyp14728-bib-0049] Leigh, R. J. , & Zee, D. S. (1999). The neurology of eye movements (3rd ed.). Oxford University Press.

[psyp14728-bib-0050] Lenth, R. (2024). Emmeans: Estimated marginal means, aka least‐squares means. R Package Version 1.10.1. https://rvlenth.github.io/emmeans/

[psyp14728-bib-0051] Majid, D. S. A. , Cai, W. , George, J. S. , Verbruggen, F. , & Aron, A. R. (2012). Transcranial magnetic stimulation reveals dissociable mechanisms for global versus selective corticomotor suppression underlying the stopping of action. Cerebral Cortex, 22(2), 363–371. 10.1093/cercor/bhr112 21666129 PMC3256406

[psyp14728-bib-0052] MathWorks . (2021). Matlab R2021b [Computer software].

[psyp14728-bib-0053] Montenegro, S. M. , & Edelman, J. A. (2019). Impact of task‐specific training on saccadic eye movement performance. Journal of Neurophysiology, 122(4), 1661–1674. 10.1152/jn.00020.2019 31461366 PMC6843085

[psyp14728-bib-0054] Munoz, D. P. , & Everling, S. (2004). Look away: The anti‐saccade task and the voluntary control of eye movement. Nature Reviews Neuroscience, 5(3), 218–228. 10.1038/nrn1345 14976521

[psyp14728-bib-0055] Nambu, A. , Tokuno, H. , & Takada, M. (2002). Functional significance of the cortico–subthalamo–pallidal ‘hyperdirect’ pathway. Neuroscience Research, 43(2), 111–117. 10.1016/S0168-0102(02)00027-5 12067746

[psyp14728-bib-0056] Novembre, G. , Pawar, V. M. , Bufacchi, R. J. , Kilintari, M. , Srinivasan, M. , Rothwell, J. C. , Haggard, P. , & Iannetti, G. D. (2018). Saliency detection as a reactive process: Unexpected sensory events evoke corticomuscular coupling. The Journal of Neuroscience, 38(9), 2385–2397. 10.1523/JNEUROSCI.2474-17.2017 29378865 PMC5830523

[psyp14728-bib-0057] Nuthmann, A. , Vitu, F. , Engbert, R. , & Kliegl, R. (2016). No evidence for a saccadic range effect for visually guided and memory‐guided saccades in simple saccade‐targeting tasks. PLoS One, 11(9), 1–27. 10.1371/journal.pone.0162449 PMC5033472 27658191

[psyp14728-bib-0058] Ouerfelli‐Ethier, J. , Elsaeid, B. , Desgroseilliers, J. , Munoz, D. P. , Blohm, G. , & Khan, A. Z. (2018). Anti‐saccades predict cognitive functions in older adults and patients with Parkinson's disease. PLoS One, 13(11), e0207589. 10.1371/journal.pone.0207589 30485332 PMC6261587

[psyp14728-bib-0059] Parmentier, F. B. R. (2014). The cognitive determinants of behavioral distraction by deviant auditory stimuli: A review. Psychological Research, 78(3), 321–338. 10.1007/s00426-013-0534-4 24363092

[psyp14728-bib-0060] Parmentier, F. B. R. , Elsley, J. V. , Andrés, P. , & Barceló, F. (2011). Why are auditory novels distracting? Contrasting the roles of novelty, violation of expectation and stimulus change. Cognition, 119(3), 374–380. 10.1016/j.cognition.2011.02.001 21382615

[psyp14728-bib-0061] Pelli, D. G. (1997). The VideoToolbox software for visual psychophysics: Transforming numbers into movies. Spatial Vision, 10(4), 437–442. 10.1163/156856897X00366 9176953

[psyp14728-bib-0062] Petrovsky, N. , Ettinger, U. , Quednow, B. B. , Landsberg, M. W. , Drees, J. , Lennertz, L. , Frommann, I. , Heilmann, K. , Sträter, B. , Kessler, H. , Dahmen, N. , Mössner, R. , Maier, W. , & Wagner, M. (2013). Nicotine enhances antisaccade performance in schizophrenia patients and healthy controls. International Journal of Neuropsychopharmacology, 16(7), 1473–1481. 10.1017/S1461145713000011 23399382

[psyp14728-bib-0063] Pierce, J. E. , & McDowell, J. E. (2016). Effects of preparation time and trial type probability on performance of anti‐ and pro‐saccades. Acta Psychologica, 164, 188–194. 10.1016/j.actpsy.2016.01.013 26829023

[psyp14728-bib-0064] Pratt, J. , & Trottier, L. (2005). Pro‐saccades and anti‐saccades to onset and offset targets. Vision Research, 45(6), 765–774. 10.1016/j.visres.2004.05.019 15639503

[psyp14728-bib-0065] R Core Team . (2024). R: A language and environment for statistical computing (4.31). R Foundation for Statistical Computing. http://www.r‐project.org/

[psyp14728-bib-0066] Reingold, E. M. , & Stampe, D. M. (2000). Saccadic inhibition and gaze contingent research paradigms. In A. Kenedy , R. Radach , D. Heller , & J. Pynte (Eds.), Reading as a perceptual process (pp. 119–145). Elsevier. 10.1016/B978-008043642-5/50008-5

[psyp14728-bib-0067] Reingold, E. M. , & Stampe, D. M. (2004). Saccadic inhibition in reading. Journal of Experimental Psychology: Human Perception and Performance, 30(1), 194–211. 10.1037/0096-1523.30.1.194 14769077

[psyp14728-bib-0068] Rettie, L. , Marsh, J. E. , Liversedge, S. P. , Wang, M. , & Degno, F. (2024). Auditory distraction during reading: Investigating the effects of background sounds on parafoveal processing. The Quarterly Journal of Experimental Psychology. 10.1177/17470218241269327 PMC12095895 39085167

[psyp14728-bib-0069] Rolfs, M. , Kliegl, R. , & Engbert, R. (2008). Toward a model of microsaccade generation: The case of microsaccadic inhibition. Journal of Vision, 8(11), 5. 10.1167/8.11.5 18831599

[psyp14728-bib-0070] Rossini, P. M. , Burke, D. , Chen, R. , Cohen, L. G. , Daskalakis, Z. , Di Iorio, R. , Di Lazzaro, V. , Ferreri, F. , Fitzgerald, P. B. , George, M. S. , Hallett, M. , Lefaucheur, J. P. , Langguth, B. , Matsumoto, H. , Miniussi, C. , Nitsche, M. A. , Pascual‐Leone, A. , Paulus, W. , Rossi, S. , … Ziemann, U. (2015). Non‐invasive electrical and magnetic stimulation of the brain, spinal cord, roots and peripheral nerves: Basic principles and procedures for routine clinical and research application. An updated report from an I.F.C.N. Committee. Clinical Neurophysiology, 126(6), 1071–1107. 10.1016/j.clinph.2015.02.001 25797650 PMC6350257

[psyp14728-bib-0071] Schröger, E. (1996). A neural mechanism for involuntary attention shifts to changes in auditory stimulation. Journal of Cognitive Neuroscience, 8(6), 527–539. 10.1162/jocn.1996.8.6.527 23961983

[psyp14728-bib-0072] Schröger, E. , & Wolff, C. (1998). Attentional orienting and reorienting is indicated by human event‐ related brain potentials. Neuroreport, 9(15), 3355–3358. 10.1097/00001756-199810260-00003 9855279

[psyp14728-bib-0073] Sokolov, E. N. (1963). Higher nervous functions: The orienting reflex. Annual Review of Physiology, 25(1), 545–580. 10.1146/annurev.ph.25.030163.002553 13977960

[psyp14728-bib-0074] Sokolov, E. N. (2001). Orienting response. In N. J. Smelser & P. B. Baltes (Eds.), International encyclopedia of the Social & Behavioral Sciences (pp. 10978–10981). Elsevier Science Ltd. 10.1016/B0-08-043076-7/03536-1

[psyp14728-bib-0075] Tatz, J. R. , Mather, A. , & Wessel, J. R. (2023). β‐Bursts over frontal cortex track the surprise of unexpected events in auditory, visual, and tactile modalities. Journal of Cognitive Neuroscience, 35(3), 485–508. 10.1162/jocn_a_01958 36603039 PMC9894628

[psyp14728-bib-0076] Valsecchi, M. , & Turatto, M. (2009). Microsaccadic responses in a bimodal oddball task. Psychological Research, 73(1), 23–33. 10.1007/s00426-008-0142-x 18320216

[psyp14728-bib-0077] Vasilev, M. R. , Lowman, M. , Bills, K. , Parmentier, F. B. R. , & Kirkby, J. A. (2023). Unexpected sounds inhibit the movement of the eyes during reading and letter scanning. Psychophysiology, 60(12), 1–19. 10.1111/psyp.14389 37448357

[psyp14728-bib-0078] Vasilev, M. R. , Parmentier, F. B. , Angele, B. , & Kirkby, J. A. (2019). Distraction by deviant sounds during reading: An eye‐movement study. Quarterly Journal of Experimental Psychology, 72(7), 1863–1875. 10.1177/1747021818820816 PMC6613176 30518304

[psyp14728-bib-0079] Vasilev, M. R. , Parmentier, F. B. , & Kirkby, J. A. (2021). Distraction by auditory novelty during reading: Evidence for disruption in saccade planning, but not saccade execution. Quarterly Journal of Experimental Psychology, 74(5), 826–842. 10.1177/1747021820982267 PMC8054167 33283659

[psyp14728-bib-0080] Walker, R. , Deubel, H. , Schneider, W. X. , & Findlay, J. M. (1997). Effect of remote distractors on saccade programming: Evidence for an extended fixation zone. Journal of Neurophysiology, 78(2), 1108–1119. 10.1152/jn.1997.78.2.1108 9307138

[psyp14728-bib-0081] Watanabe, M. , & Munoz, D. P. (2011). Probing basal ganglia functions by saccade eye movements. European Journal of Neuroscience, 33(11), 2070–2090. 10.1111/j.1460-9568.2011.07691.x 21645102

[psyp14728-bib-0082] Wenban‐Smith, M. G. , & Findlay, J. M. (1991). Express saccades: Is there a separate population in humans? Experimental Brain Research, 87(1), 218–222. 10.1007/BF00228523 1756828

[psyp14728-bib-0083] Wessel, J. R. (2017). Perceptual surprise aides inhibitory motor control. Journal of Experimental Psychology: Human Perception and Performance, 43(9), 1585–1593. 10.1037/xhp0000452 28557496

[psyp14728-bib-0084] Wessel, J. R. (2018a). An adaptive orienting theory of error processing. Psychophysiology, 55(3), 1–21. 10.1111/psyp.13041 29226960

[psyp14728-bib-0085] Wessel, J. R. (2018b). Surprise: A more realistic framework for studying action stopping? Trends in Cognitive Sciences, 22(9), 741–744. 10.1016/j.tics.2018.06.005 30122169 PMC7714639

[psyp14728-bib-0086] Wessel, J. R. , & Aron, A. R. (2013). Unexpected events induce motor slowing via a brain mechanism for action‐stopping with global suppressive effects. Journal of Neuroscience, 33(47), 18481–18491. 10.1523/JNEUROSCI.3456-13.2013 24259571 PMC3834054

[psyp14728-bib-0087] Wessel, J. R. , & Aron, A. R. (2017). On the globality of motor suppression: Unexpected events and their influence on behavior and cognition. Neuron, 93(2), 259–280. 10.1016/j.neuron.2016.12.013 28103476 PMC5260803

[psyp14728-bib-0088] White, A. L. , & Rolfs, M. (2016). Oculomotor inhibition covaries with conscious detection. Journal of Neurophysiology, 116(3), 1507–1521. 10.1152/jn.00268.2016 27385794 PMC5040379

[psyp14728-bib-0089] Widmann, A. , Engbert, R. , & Schröger, E. (2014). Microsaccadic responses indicate fast categorization of sounds: A novel approach to study auditory cognition. Journal of Neuroscience, 34(33), 11152–11158. 10.1523/JNEUROSCI.1568-14.2014 25122911 PMC6705255

[psyp14728-bib-0090] Wilson, M. T. , Moezzi, B. , & Rogasch, N. C. (2021). Modeling motor‐evoked potentials from neural field simulations of transcranial magnetic stimulation. Clinical Neurophysiology, 132(2), 412–428. 10.1016/j.clinph.2020.10.032 33450564

[psyp14728-bib-0091] Zeligman, L. , & Zivotofsky, A. Z. (2017). Back to basics: The effects of block vs. interleaved trial administration on pro‐ and anti‐saccade performance. PLoS One, 12(2), 1–16. 10.1371/journal.pone.0172485 PMC5319747 28222173

[psyp14728-bib-0092] Zhao, S. , Yum, N. W. , Benjamin, L. , Benhamou, E. , Yoneya, M. , Furukawa, S. , Dick, F. , Slaney, M. , & Chait, M. (2019). Rapid ocular responses are modulated by bottom‐up‐driven auditory salience. The Journal of Neuroscience, 39(39), 7703–7714. 10.1523/JNEUROSCI.0776-19.2019 31391262 PMC6764203

