# Supplementary material for: Unexpected sounds induce a rapid inhibition of eye‐movement responses
Source: Psychophysiology. 2024 Dec 17;62(1):e14728. doi: 10.1111/psyp.14728 (PMC11652409; doi:10.1111/psyp.14728)
Supplement: Supplementary file 1 — Data S1: Modulation of novelty distraction by trial order. [file PSYP-62-e14728-s001.docx]

**Supplemental Materials**

**Modulation of Novelty Distraction by Trial Order**

There is some evidence suggesting that distraction by unexpected sounds decreases throughout the experiment (e.g., Parmentier, 2008; Wessel & Aron, 2013; Wetzel et al., 2021), presumably as the acoustic novelty of sounds wears off and participants gradually learn to expect novel sounds with a certain probability. To find out if this was the case in the present study, we conducted a post-hoc analysis, testing if the novelty distraction effect changed throughout the experiment. The trials for each task were grouped into small “blocks” of 30, which were used for the purpose of sound randomisation in the study. Each block consisted of 48 trials (8 novel and 40 standard- corresponding to one full run of all the conditions within a task) and the order of blocks corresponded to the order in which they appeared in the study.

The statistical analysis was done with Generalised Additive Mixed Models (GAMMs) using the “mgcv” R package v.1.9-1 (Wood, 2011, 2017). GAMMs are especially useful for modelling temporally correlated data (such as groups of trials that occur one after another), especially if the data exhibits potentially non-linear patterns. In GAMMs, some of the predictors are specified as “smooths”, which represent the weighted sum of cubic spline functions (Baayen et al., 2017; Sóskuthy, 2017). In this analysis, smooths were included for the random intercept of subjects, the random slope of sound for subjects, the effect of block order, the interactions between subject and block order, and the interaction between sound and block order. The remaining fixed effects were the same as LMM model in the main text (Table 2). The results from the analysis are visualised in Figure S1.

There was a significant interaction between block order and novel sounds (*edf*= 3.94, *F*(4.85)= 32.896, *p* < 0.001). As Figure S1 shows, SRTs in the novel sound condition generally tended to decrease as the experiment progressed, before reaching a plateau towards the end of the experiment. Standard sounds did not exhibit an interaction with block order (edf= 0.0009, *F*(0.001)= 0.026, *p*= 0.99), suggesting that they remained relatively constant during the experiment. In the anti-saccade task, the regression line for standard sounds was mostly flat; in the pro-saccade task, there was a small trend for SRTs to *increase* towards the end of the experiment. However, overall, the interaction between standard sounds and block order was not significant.

Looking at the difference between Novel and Standard sounds (see the right-hand plots in Figure S1), the novelty distraction effect size decreased throughout the experiment. However, the effect generally remained significant until the end of the study. A few small exceptions to this were the -150ms, -125ms, and -75 ms sound onset conditions in the pro-saccade task, where the effect was no longer significant in the last several blocks. Therefore, the present data generally supports previous studies (e.g., Parmentier, 2008; Wetzel et al., 2021) and suggests that novelty distraction tends to decrease and plateau towards the end of the experiment. Interestingly, the effect was no longer significant in some of the earliest delay conditions in the pro-saccade task towards the end of the study. This suggests that the inhibition there was weaker and/or wore off more quickly. Of course, the present study presented only a limited number of novel sounds per task (240 in total). Therefore, it is possible that the effect may also wear off in some of the remaining conditions if more testing had been done.


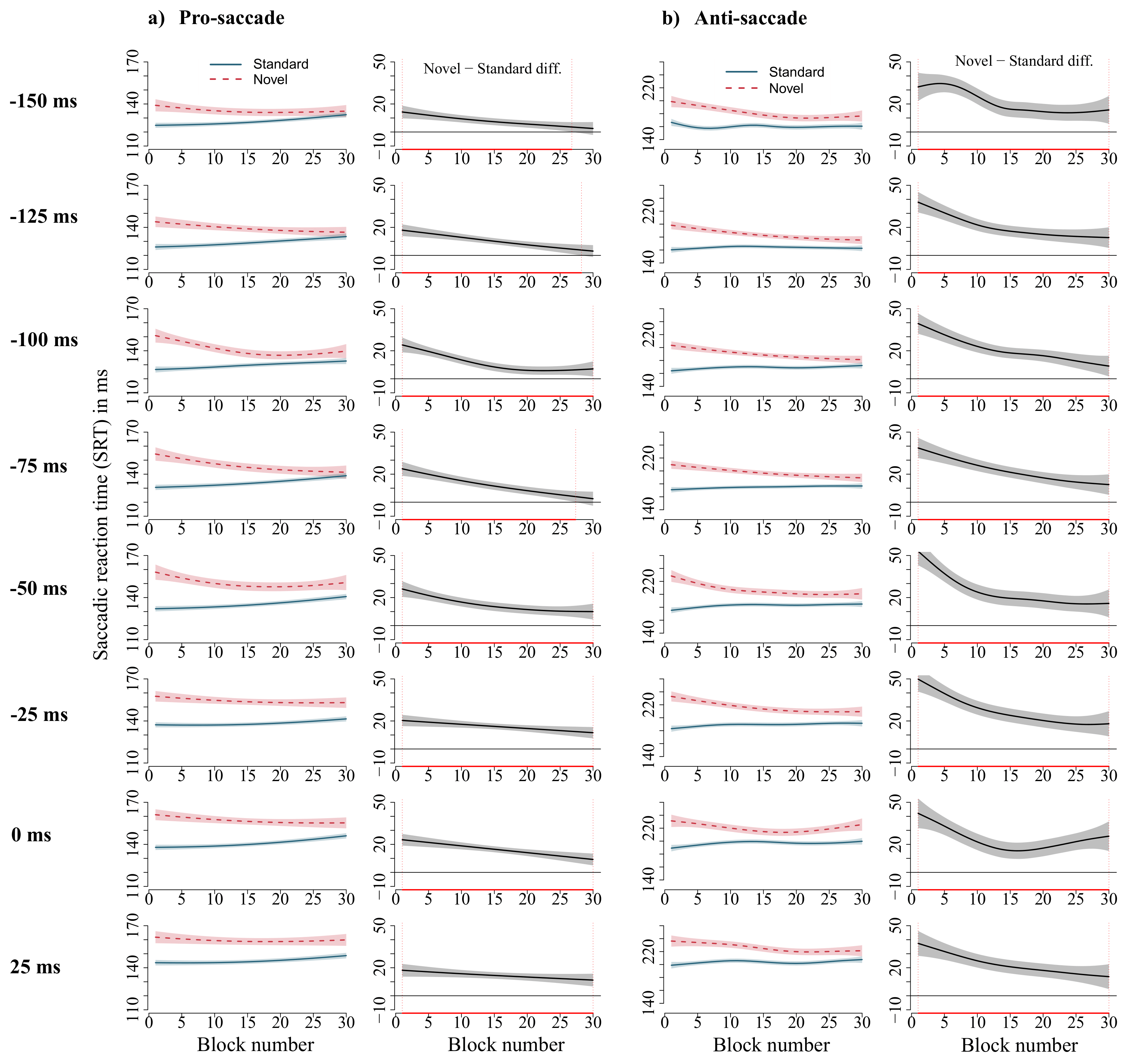


*Figure S1*. Modulation of the novelty distraction effect in the pro-saccade (panel **a**) and the anti-saccade (panel **b**) task. In each panel, the plots on the left show the saccadic reaction times (SRTs) for each sound type as a function of block number. The plots on the right in each panel show the novelty distraction effect size in SRTs (Novel- Standard difference) as a function of block number. Shading indicates 95% CIs. Note that the effect is significant if the regression line CI limits are above 0. For convenience, red colour on the x-axis indicates the block numbers during which the novelty distraction effect was significant.

**Analysis of the data with Linear and Quadratic Terms for Sound Onset**

The analyses in the main paper treated Sound Onset as a discrete predictor and compared each time interval to the next. The results indicated that the magnitude of the novelty distraction effect did not change with Sound Onset, as there were no significant two-way or three-way interactions. However, there could still be small changes that occur between the different levels that are not detected by this analysis. For this reason, we conducted post-hoc supplementary analyses where Sound Onset was treated as a continuous variable. We included both linear and quadratic terms for Sound Onset. Adding the quadratic term significantly improved the model fit for SRTs (χ^2^(4)= 52.60, p< .001) and saccade amplitude (χ^2^(4)= 38210, p< .001). For anti-saccade error rate, the model failed to converge with just a linear term for Sound Onset. Therefore, for this measure, we also report the model with both linear and quadratic terms (which did converge). The results are presented in Tables S1 and S2.

**Saccadic reaction times.** The results were generally consistent with the model reported in the main paper (see Table 2). For SRTs, novel sounds were more distracting than standard sounds (*b*= 0.048, *SE*= 0.003, *t*=16.439, *p* <0.001), and the significant Sound by Task interaction (*b*= 0.008, *SE*= 0.001, *t*= 9.464, *p* <0.001) shows that this effect was stronger in the anti-saccade task than the pro-saccade task. Sound Onset had a significant positive effect in both the linear (*b*= 28.039, *SE*= 0.343, *t*= 81.676, *p* <0.001) and quadratic terms (*b*= 0.895, *SE*= 0.343, *t*= 2.610, *p*= 0.009), with SRTs increasing as the sound was played closer in time to the target. This is again consistent with the main model in the paper. However, there was a significant Sound by Sound Onset interaction in both linear (*b*= 0.969, *SE*= 0.343, *t*= 2.823, *p*= 0.005) and quadratic terms (*b*= -1.067, *SE*= 0.343, *t*= -3.108, *p*= 0.002). As Figure S2 shows, the difference between novel and standard sounds increased slightly as the sound was played closer in time to the target, before reaching a plateau around -50 ms. The three-way interaction between Sound, Task and Sound Onset was significant in the linear (*b*= -1.724, *SE*= 0.343, *t*= -5.023, *p* <0.001), but not the quadratic term (*b*= 0.165, *SE*= 0.343, *t*= 0.480, *p*= 0.631). As Figure S2 shows, there was a trend for the difference between novel and standard sounds to increase more strongly with sound onset in the pro-saccade compared to the anti-saccade task. In summary, the results confirm the main model in the paper, but also suggest that inhibition of SRTs by novel sounds was slightly weaker in the first few onset conditions. This trend was more pronounced in the pro-saccade task.


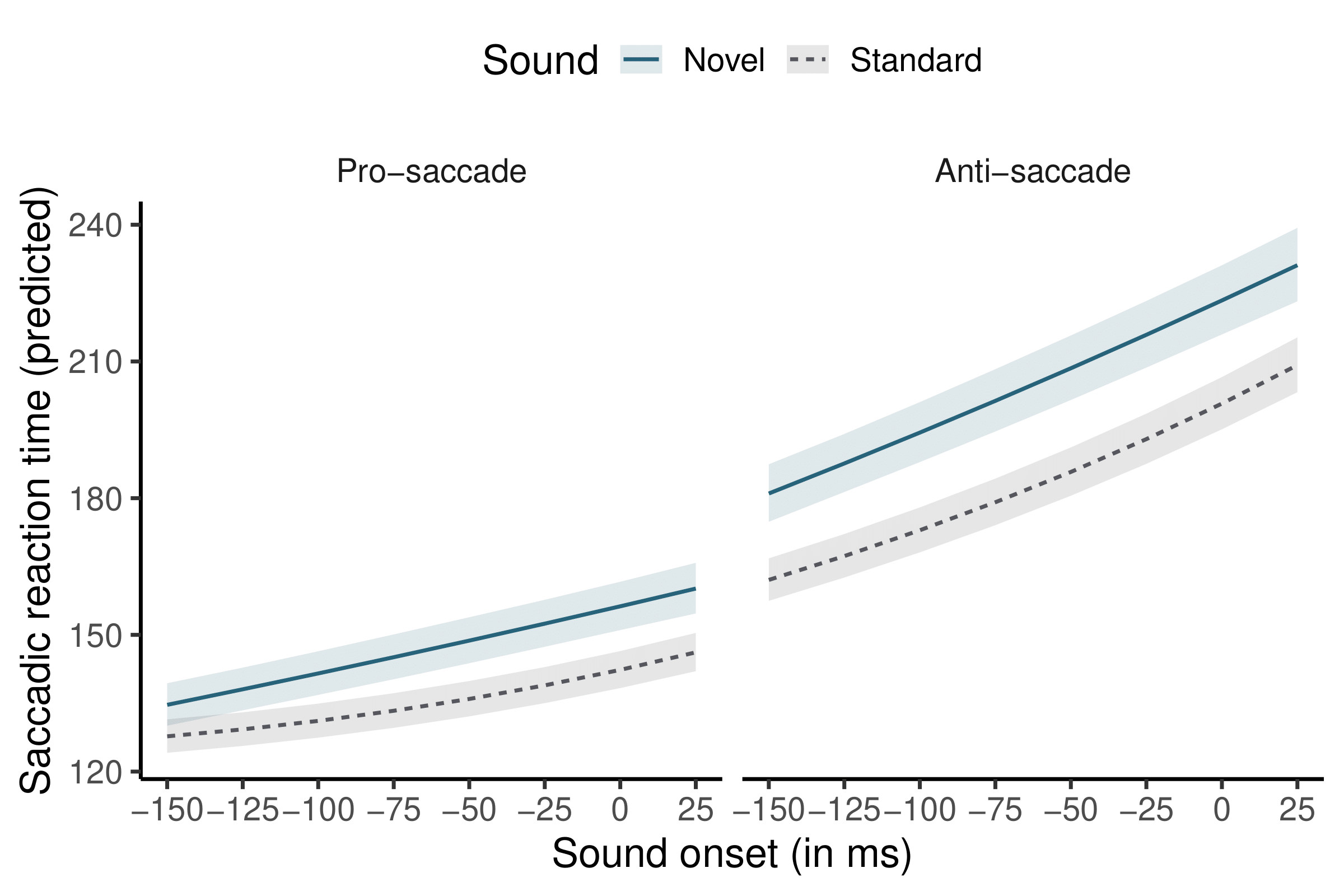


*Figure S2*. Predicted saccadic reaction times from an LMM model that treats sound onset as a continuous variable and includes both linear and quadratic terms for it. Shading indicates 95% CIs.

Table S1

*Linear Mixed Effect Results for Saccadic Reaction Time and Saccade Amplitude, Treating Sound Onset as a Continuous Variable (using Linear and Quadratic Terms)*

|  | **log(Saccadic reaction time)** | | | | **Saccade amplitude** | | | |
| --- | --- | --- | --- | --- | --- | --- | --- | --- |
| *Predictors* | *Estimate* | *std. Error* | *Statistic* | *p* | *Estimate* | *std. Error* | *Statistic* | *p* |
| (Intercept) | 5.107 | 0.016 | 327.772 | **<0.001** | 9.722 | 0.164 | 59.324 | **<0.001** |
| Sound | 0.048 | 0.003 | 16.439 | **<0.001** | 0.045 | 0.008 | 5.651 | **<0.001** |
| Task | 0.158 | 0.001 | 194.209 | **<0.001** | -0.271 | 0.148 | -1.836 | 0.066 |
| Onset [Linear] | 28.039 | 0.343 | 81.676 | **<0.001** | -8.502 | 3.410 | -2.494 | **0.013** |
| Onset [Quadratic] | 0.895 | 0.343 | 2.610 | **0.009** | -13.335 | 3.408 | -3.912 | **<0.001** |
| Sound × Task | 0.008 | 0.001 | 9.464 | **<0.001** | -0.031 | 0.008 | -3.813 | **<0.001** |
| Sound × Onset [Linear] | 0.969 | 0.343 | 2.823 | **0.005** | 12.577 | 3.410 | 3.689 | **<0.001** |
| Sound × Onset [Quadratic] | -1.067 | 0.343 | -3.108 | **0.002** | 1.138 | 3.408 | 0.334 | 0.738 |
| Task × Onset [Linear] | 6.642 | 0.343 | 19.350 | **<0.001** | -7.502 | 3.410 | -2.200 | 0.028 |
| Task × Onset [Quadratic] | -0.271 | 0.343 | -0.791 | 0.429 | -1.547 | 3.408 | -0.454 | 0.650 |
| Sound× Task × Onset [Linear] | -1.724 | 0.343 | -5.023 | **<0.001** | 2.705 | 3.410 | 0.793 | 0.428 |
| Sound × Task × Onset [Quadratic] | 0.165 | 0.343 | 0.480 | 0.631 | 1.759 | 3.408 | 0.516 | 0.606 |
| **Random Effects** | | | | | | | | |
| σ^2^ | 0.065 | | | | 6.457 | | | |
| τ_00_ | 0.017 _sub_ | | | | 1.929 _sub_ | | | |
| τ_11_ | 0.001 _sub.sound_ | | | | 1.568 _sub.task_ | | | |
| ρ_01_ | 0.454 _sub_ | | | | 0.906 _sub_ | | | |
| ICC | 0.197 | | | | 0.340 | | | |
| N | 72 _sub_ | | | | 72 _sub_ | | | |
| Observations | 180818 | | | | 180818 | | | |
| Marginal R^2^ / Conditional R^2^ | 0.262 / 0.408 | | | | 0.007 / 0.345 | | | |

*Note*: Statistically significant p-values are formatted in **bold**. A Bonferroni correction was applied and the significance threshold was 0.05/3= 0.016.

**Saccade amplitude**. Similar to the main model in the paper, novel sounds led to significantly longer saccades compared to standard sounds (*b*= 0.045, *SE*= 0.008, *t*= 5.651, *p* <0.001). Additionally, the effect of Sound Onset was significant in both the linear (*b*= -8.502, *SE*= 3.410, *t*= -2.494, *p*= 0.013) and quadratic (*b*= -13.335, *SE*= 3.408, *t*= -3.912, *p* <0.001) terms. As Figure S3 shows, there was a small decrease in saccade amplitudes in the last few sound onset conditions. The interaction between Sound and Task was also significant (*b*= -0.031, *SE*= 0.008, *t*= -3.813 *p* <0.001). This was again due to the difference between novel and standard sounds being significant in the pro-saccade, but not in the anti-saccade task. Finally, the interaction between Sound and Sound Onset was significant for the linear (*b*= 12.577, *SE*= 3.410, *t*= 3.689, *p* <0.001), but not for the quadratic term (*b*= 1.138, *SE*= 3.408, *t*= 0.334, *p*= 0.738). There was a trend for the difference between standard and novel sounds to increase in size in the pro-saccade, but not in the anti-saccade task. Therefore, the results were consistent with the model reported in the main paper, but further suggest that the difference in saccade length between the sounds in the pro-saccade task increased with sound onset.


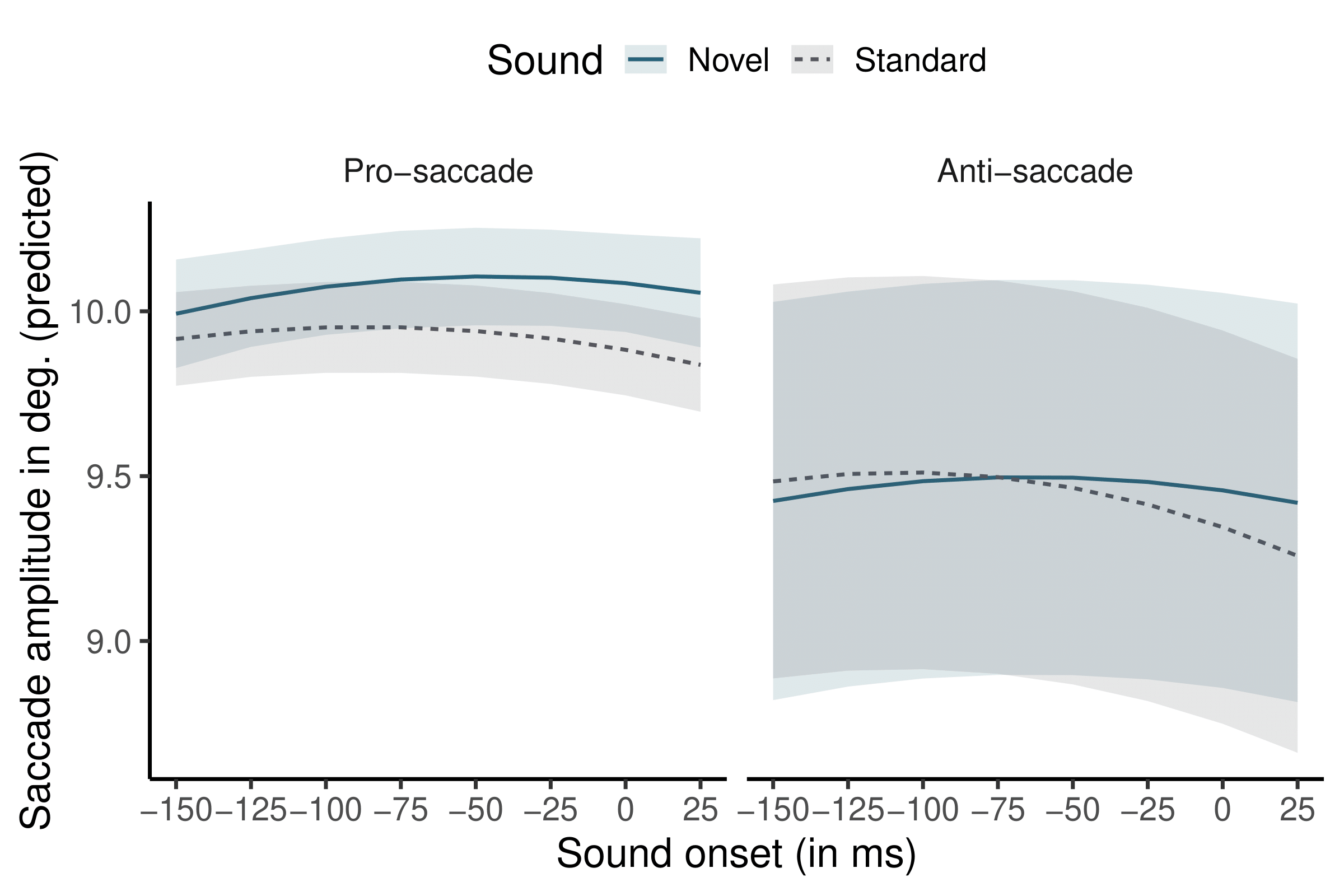


*Figure S3*. Predicted saccade amplitudes from an LMM model that treats sound onset as a continuous variable and includes both linear and quadratic terms for it. Shading indicates 95% CIs.

**Anti-saccade error rate.** The GLMM results are shown in Table S2. Consistent with the main model in the paper, novel sounds led to significantly lower error rates in the anti-saccade task compared to standard sounds (*b*= -0.245, *SE*= 0.012, *z*= -20.086, *p* <0.001). Sound Onset also had a significant effect on error rates in both the linear (*b*= -48.776, *SE*= 0.904, *z*= -53.943, *p* <0.001) and quadratic terms (*b*= 2.288, *SE*= 0.836, *z*= 2.736, *p*= 0.006). As Figure S4 shows, error rates decreased as the sound was played closer to the target. Therefore, while some of the successive Sound Onset differences were not significant in the main model in the paper, this analysis shows that there was an overall trend for error rates to decrease with Sound Onset. Finally, the interaction between Sound and Sound Onset was also significant in both the linear (*b*= -10.754, *SE*= 0.890, *z*= -12.086, *p* <0.001) and quadratic terms (*b*= 4.985, *SE*= 1.031, *z*= 4.835, *p* <0.001). As Figure 4 shows, this was due the difference between novel and standard sounds increasing with Sound Onset, until reaching a plateau around -50 ms. In summary, the results confirm the main paper model, but also suggest that the decrease in error rates by novel sounds was smallest in the first few onset conditions and generally increased when the sound was played closer to the target. Interestingly, this trend closely resembles the pattern observed in the SRT data.

**
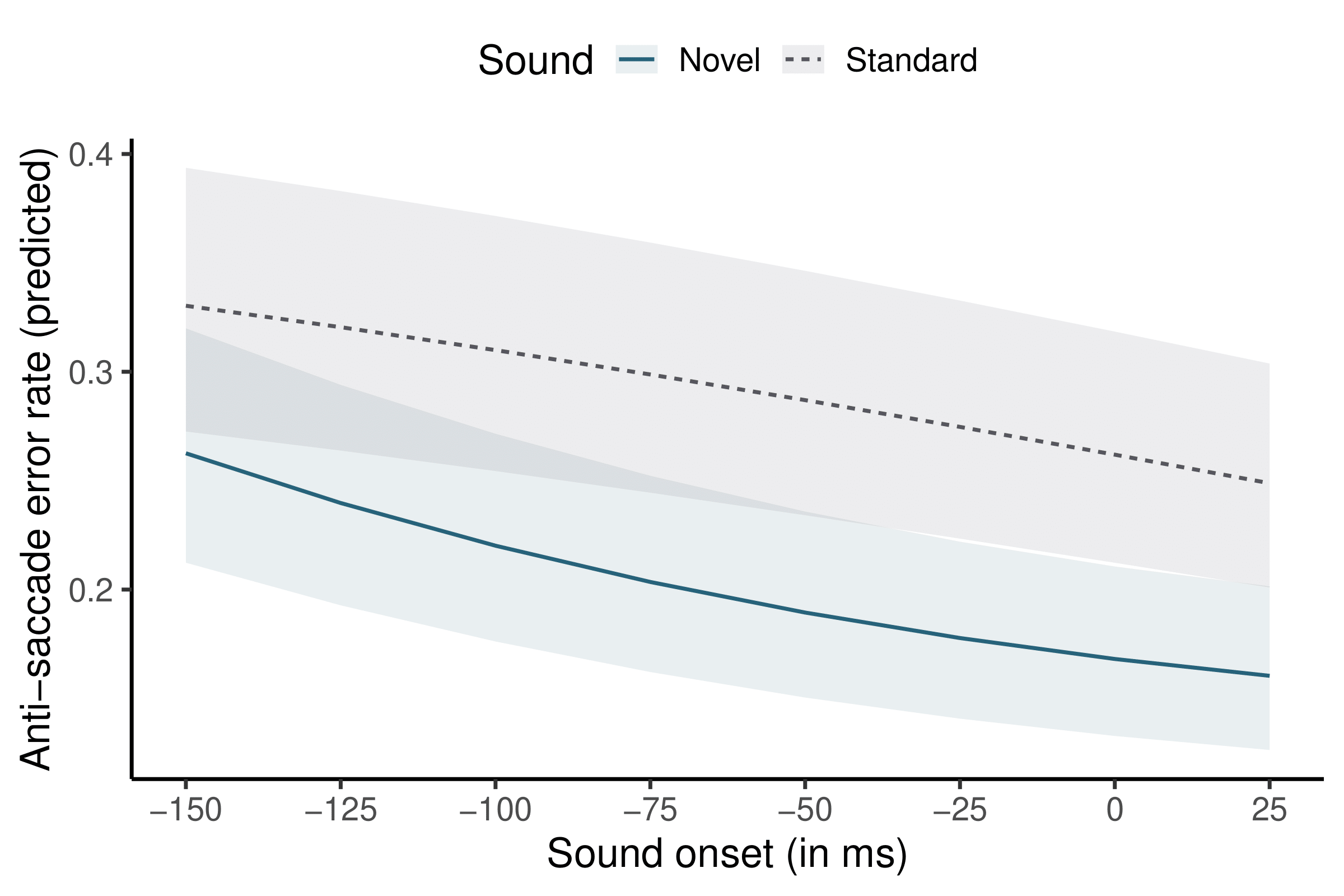
**

*Figure S4*. Predicted anti-saccade error rates from a GLMM model that treats sound onset as a continuous variable and includes both linear and quadratic terms for it. Shading indicates 95% CIs.

Table S2

*Linear Mixed Effect Results for Anti-saccade Error Rate, Treating Sound Onset as a Continuous Variable (using Linear and Quadratic Terms)*

|  | **Anti-saccade error rate** | | | |
| --- | --- | --- | --- | --- |
| *Predictors* | *Estimate* | *std. Error* | *z statistic* | *p* |
| (Intercept) | -1.136 | 0.140 | -8.090 | **<0.001** |
| Sound | -0.245 | 0.012 | -20.086 | **<0.001** |
| Onset [Linear] | -48.776 | 0.904 | -53.943 | **<0.001** |
| Onset [Quadratic] | 2.288 | 0.836 | 2.736 | **0.006** |
| Sound × Onset [Linear] | -10.754 | 0.890 | -12.086 | **<0.001** |
| Sound × Onset [Quadratic] | 4.985 | 1.031 | 4.835 | **<0.001** |
| **Random Effects** | | | | |
| σ^2^ | 3.290 | | | |
| τ_00_ _sub_ | 1.444 | | | |
| ICC | 0.305 | | | |
| N _sub_ | 72 | | | |
| Observations | 85699 | | | |
| Marginal R^2^ / Conditional R^2^ | 0.011 / 0.313 | | | |

*Note*: Statistically significant p-values are formatted in **bold**. A Bonferroni correction was applied and the significance threshold was 0.05/3= 0.016.

**Effect of Sound on Eye Velocity in Subsequent Saccade**

The analysis of saccade amplitudes in the main paper suggested that novel sounds have a limited effect on saccade execution. To further explore this, we analysed average saccade velocity as a function of the time between playing the sound and the onset of the next saccade (rather than the onset of the visual target). This may give more sensitive information about the timeline of potential effects compared to the experimental sound onset condition. The results are visualised in Figure S5 and the LMM results are presented in Table S3.


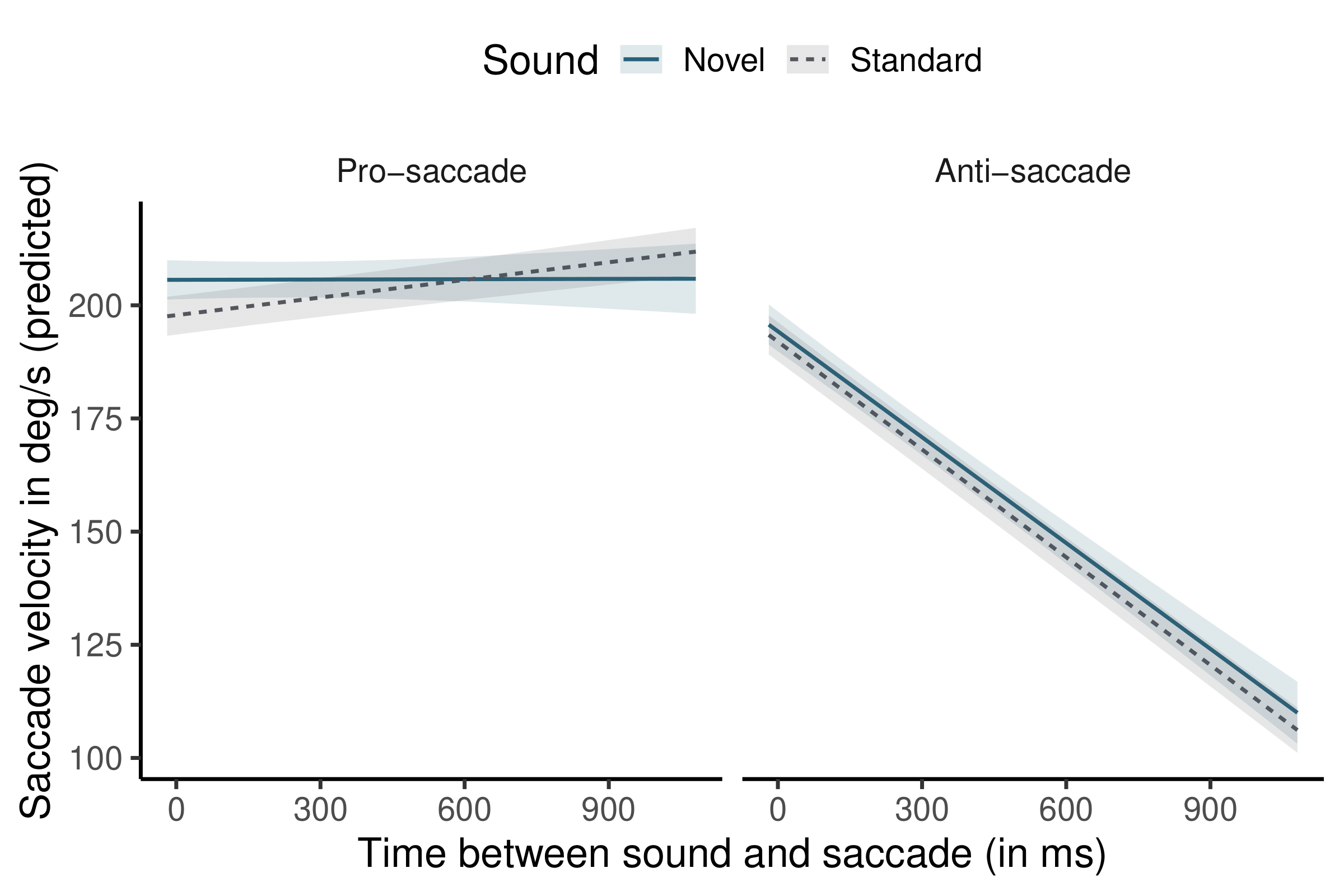


*Figure S5*. Average saccade velocity in the two tasks and sound conditions as a function of the time duration between playing the sound and the onset of the saccade. Estimates are derived from the LMM model predictions. Note that the duration was centred at 0 in the analysis and back-transformed here for visualisation purposes. Shading indicates 95% CIs.

The results followed the saccade amplitude data closely. Novel sounds led to significantly higher average velocity compared to standard sounds (*b*= 1.868, *SE*= 0.327, *t*= 5.715, *p* <0.001). Additionally, the pro-saccade task led to higher velocities compared to the anti-saccade task (*b*= -14.163, SE= 0.117, t= -120.745, p <0.001). There was also a significant interaction between Sound and Task (*b*= -0.561, SE= 0.117, t= -4.790, p <0.001). Similar to the saccade amplitude data, this was again due to a bigger Sound effect in the pro-saccade task compared to the anti-saccade task. Therefore, these results indicate a close correspondence between saccade amplitudes and saccade velocity. This is not surprising as the two measures are highly correlated with each other (Bahill et al., 1975).

Furthermore, longer durations between sound onset and saccade onset were associated with lower saccade velocities. However, there was a significant Time to saccade by Task interaction (*b*= -0.043, *SE*= = 0.001, *t*= -29.851, *p* <0.001). As Figure S5 shows, velocities decreased more strongly with greater Time to saccade in the anti-saccade task compared to the pro-saccade task. Finally, the three-way interaction between Sound, Task, and Time to saccade was also significant (*b*= 0.004, *SE*= 0.001, *t*= 2.484, *p*= 0.013). This is again illustrated in Figure S5, as velocities following standard sounds tended to increase in the pro-saccade task while velocities following novel sounds remained constant. This was in contrast to the anti-saccade task, where no such modulation was evident. Therefore, these results confirm the main analysis with saccade amplitudes. They also illustrate that the modulation of saccade execution in the pro-saccade task is mostly driven by changes following the standard sound. In summary, these results confirm previous reports that unexpected sounds have no notable effect on saccade execution (Vasilev et al., 2021).

Table S3

*LMM Results for Average Saccade Velocity, as Predicted by Sound, Task, and Time from Sound Onset until Saccade Onset*

|  | **Average saccade velocity** | | | |
| --- | --- | --- | --- | --- |
| *Predictors* | *Estimate* | *std. Error* | *Statistic* | *p* |
| (Intercept) | 189.083 | 2.060 | 91.780 | **<0.001** |
| Sound | 1.868 | 0.327 | 5.715 | **<0.001** |
| Task | -14.163 | 0.117 | -120.745 | **<0.001** |
| Time to saccade | -0.036 | 0.001 | -24.037 | **<0.001** |
| Sound × Task | -0.561 | 0.117 | -4.790 | **<0.001** |
| Sound × Time to saccade | -0.003 | 0.001 | -1.895 | 0.058 |
| Task × Time to saccade | -0.043 | 0.001 | -29.851 | **<0.001** |
| Sound × Task × Time to saccade | 0.004 | 0.001 | 2.484 | **0.013** |
| **Random Effects** | | | | |
| σ^2^ | 1120.283 | | | |
| τ_00_ _sub_ | 304.613 | | | |
| τ_11_ _sub.sound_ | 6.711 | | | |
| ρ_01_ _sub_ | -0.262 | | | |
| ICC | 0.226 | | | |
| N _sub_ | 72 | | | |
| Observations | 180813 | | | |
| Marginal R^2^ / Conditional R^2^ | 0.142 / 0.336 | | | |

Note

References

Baayen, H., Vasishth, S., Kliegl, R., & Bates, D. (2017). The cave of shadows: Addressing the human factor with generalized additive mixed models. *Journal of Memory and Language*, *94*, 206–234. https://doi.org/10.1016/j.jml.2016.11.006

Bahill, A. T., Clark, M. R., & Stark, L. (1975). The main sequence, a tool for studying human eye movements. *Mathematical Biosciences*, *24*(3–4), 191–204. https://doi.org/10.1016/0025-5564(75)90075-9

Parmentier, F. B. R. (2008). Towards a cognitive model of distraction by auditory novelty: The role of involuntary attention capture and semantic processing. *Cognition*, *109*(3), 345–362. https://doi.org/10.1016/j.cognition.2008.09.005

Sóskuthy, M. (2017). *Generalised additive mixed models for dynamic analysis in linguistics: A practical introduction*. http://eprints.whiterose.ac.uk/113858/2/1703_05339v1.pdf

Vasilev, M. R., Parmentier, F. B., & Kirkby, J. A. (2021). Distraction by auditory novelty during reading: Evidence for disruption in saccade planning, but not saccade execution. *Quarterly Journal of Experimental Psychology*, *74*(5), 826–842. https://doi.org/10.1177/1747021820982267

Wessel, J. R., & Aron, A. R. (2013). Unexpected events induce motor slowing via a brain mechanism for action-stopping with global suppressive effects. *Journal of Neuroscience*, *33*(47), 18481–18491. https://doi.org/10.1523/JNEUROSCI.3456-13.2013

Wetzel, N., Widmann, A., & Scharf, F. (2021). Distraction of attention by novel sounds in children declines fast. *Scientific Reports*, 1–14. https://doi.org/10.1038/s41598-021-83528-y

Wood, S. N. (2011). Fast Stable Restricted Maximum Likelihood and Marginal Likelihood Estimation of Semiparametric Generalized Linear Models. *Journal of the Royal Statistical Society Series B: Statistical Methodology*, *73*(1), 3–36. https://doi.org/10.1111/j.1467-9868.2010.00749.x

Wood, S. N. (2017). *Generalized additive models: An introduction with R* (2nd ed.). Chapman and Hall/CRC.
